# A pair-conformation-dependent scoring function for evaluating 3D RNA-protein complex structures

**DOI:** 10.1371/journal.pone.0174662

**Published:** 2017-03-30

**Authors:** Haotian Li, Yangyu Huang, Yi Xiao

**Affiliations:** Biomolecular Physics and Modeling Group, School of Physics, Huazhong University of Science and Technology, Wuhan, Hubei, China; University of Michigan, UNITED STATES

## Abstract

Computational prediction of RNA-protein complex 3D structures includes two basic steps: one is sampling possible structures and another is scoring the sampled structures to pick out the correct one. At present, constructing accurate scoring functions is still not well solved and the performances of the scoring functions usually depend on used benchmarks. Here we propose a pair-conformation-dependent scoring function, 3dRPC-Score, for 3D RNA-protein complex structure prediction by considering the nucleotide-residue pairs having the same energy if their conformations are similar, instead of the distance-only dependence of the most existing scoring functions. Benchmarking shows that 3dRPC-Score has a consistent performance in three test sets.

## Introduction

RNA-Protein interactions are widespread in cells [1–7]. For understanding these interactions, 3D structures of RNA-protein complexes are necessary. However, at present it’s still difficult to solve 3D RNA-protein complex structures by experimental methods [8, 9]. The number of 3D protein/RNA monomer structures is much larger than that of 3D RNA-protein complex structures in Protein Data Bank (PDB) [10]. So some computational methods to predict the 3D structures of RNA-protein complexes from protein and RNA monomer structures have been developed [11–17].

Prediction of 3D RNA-protein complex structures includes two key steps: sampling and scoring. Sampling means constructing possible models of 3D complex structure (decoys) from 3D monomer structures. Scoring is picking out the correct models from the decoys by using a scoring function. For RNA-protein complex structure prediction, some scoring functions have been proposed, such as DARS-RNP and QUASI-RNP [12], DECK-RP[15], ITScore-PR [18] and so on [14, 19–25]. The scoring function DARS-RNP and QUASI-RNP [12] not only depended on the distance between residue and nucleotide of a pair but also their relative orientation. The scoring function ITScore-PR is a distance-dependent statistical potential and uses an iterative method to avoid the problems of the reference states. The scoring function DECK-RP is also a distance-dependent statistical potential and is included in our program of 3D RNA-protein complex structure prediction 3dRPC [15]. DECK-RP was constructed based on the protein-protein scoring function DECK [26] and it also combined the advantages of the two existing potentials, Li’s potential [14] and DARS-RNP [12], including the environment (secondary structure) information of the nucleotide-residue pairs. DECK-RP also used a reference state with a decoy-based component and a mol-fraction corrected component. Although these improvements, the distance-dependent statistical potentials are not very accurate in the sense that the energy of an amino acid pair or an amino acid-nucleotide pair cannot be accurately defined by only using the relative distance between the partners of the pair. Both the relative distance and orientation of them, i.e., the pair conformation, are needed (see more discussions in the discussion section). Therefore, it is more reasonable to use the conformations of an amino acid pair or an amino acid-nucleotide pair as a metric to construct its statistical potential.

Here we propose a novel score function 3dRPC-Score based on statistical potential that uses the conformations of nucleotide-residue pairs as statistical variables, i.e., the energy of a nucleotide-residue pair depends on its conformations [27]. The types of conformations of a nucleotide-residue pair are classified by using the relative RMSD (Root Mean Square Deviation) between the conformations. On the one hand, 3dRPC-Score is different from widely used distance-dependent statistical potential like DECK-RP and ITScore-PR; On the other hand, it uses only one variable (RMSD) instead of two variables (distance and orientation) as used in DARS-RNP and QUASI-RNP [12]. 3dRPC-Score are tested on three RNA-protein unbound docking benchmarks provided by Huang and Zou [18, 28] and Perez Cano et al [29], and it gives a consistent performance on these different test sets.

## Method

### Training and testing datasets

#### Training set I

A decoy dataset is needed to train the parameter in our statistical potential. For this we used the bound-docking decoy set provided by Huang et al[15]. From it we removed the structures that exist in test sets and 96 native RNA-protein complexes are remained in the training set (S1 Table). For each native protein-RNA complex in the training set, 1000 bound-docking decoy structures were created by using RPDOCK [15].

#### Test set

We test 3dRPC-Score in three RNA-protein unbound docking datasets. The first test set was constructed by Huang and Zou [28]. For each target, 1000 decoy structures were created by RPDOCK [15]. There are 72 RNA-protein complexes in this test set, including 49 easy targets, 16 medium targets and 7 difficult targets, which are classified based on the interface RMSD (IRMSD) and the percentage of native contacts in the unbound structures [28] (S2 Table). The second test set was also that constructed by Huang and Zou [28] but the 1000 decoy structures of each target were generated by ZDOCK [30]. The third test set is from Perez Cano et al [29]. From it we removed the same structures in training sets and the targets containing modeled structures and finally 64 targets remained in it (S3 Table). For each target in this test set, 1000 decoy structures were generated by RPDOCK [15]. Based on the IRMSD between native and unbound structures [29], the targets of this test set can be divided into three categories: easy (IRMSD<2.5Å), medium (2.5Å≤IRMSD≤5Å) and difficult (IRMSD>5Å). There are 46 easy targets, 11 medium targets and 7 difficult targets. Using this standard to Zou’s test set, it has 58 easy targets, 9 medium targets and 5 difficult targets. Therefore, Perez Cano’s test set is more difficult than Zou’s in this sense.

### Extracting standard nucleotide-residue pairs

3dRPC-Score is a statistical potential that depends on the conformations of nucleotide-residue pairs. A nucleotide-residue pair is defined as a pair of nucleotide and residue whose center distance is less than 10 Å. The conformations of various nucleotide-residue pairs were extracted from the structures of ribosomes. For this we selected the X-ray crystal structures of ribosomes from PDB that have a resolution less than 3 Å and the sequence identity is <30% (S4 Table).

There are 80 (20×4) types of nucleotide-residue pairs since there are 20 kinds of amino acids and four kinds of nucleotides. By using the k-means clustering method, the conformations of each nucleotide-residue pair are clustered into 10 classes based on their relative RMSDs. Thus, we get 800 conformational classes. The pair conformations in each class are similar to each other (see Discussions) and so we use the center conformation of each class as standard nucleotide-residue pair to represent the pairs in this class.

### Building the statistical potential

The pairs in each class have similar conformations and so they can be considered to have the same energy. Therefore, the statistical potential can be written as:

$$E_{ij}\left(C\right)=-\ln\left(\frac{P_{ij}\left(C\right)}{P_{i}P_{j}*P_{v}}\right)$$ (1)

In which *P*_*ij*_(*C*) is the occurrence probability of the pair of *i*-type nucleotide and *j*-type residue in class C; *P*_*i*_(*P*_*j*_) is the probability of nucleotide *i* (residue j) in the interface. *P*_*ij*_(*C*), *P*_*i*_ and *P*_*j*_ are from the statistics of the selected ribosome structures. We determine whether a nucleotide/residue is in the interface based on the solvent accessible area. If the solvent accessible area of a nucleotide/residue is different for monomer and complex, the nucleotide/residue is in the interface. And we used AMBER14 to calculate the solvent accessible area[31]. *P*_*v*_ is the probability of class C in the whole conformational space of nucleotide-residue pairs in ideal state. In ideal state, each class of the nucleotide-residue pairs has the same probability in conformation space and so we have from Eq (1)

$$E_{ij}\left(C\right)=-\ln\left(\frac{P_{ij}\left(C\right)}{P_{i}P_{j}}\right)+constant$$ (2)

where the constant = *lnP*_*v*_ is less than 0 since *P*_*v*_ is a constant and less than 1.

### Training the constant in the scoring function

**Fig 1:**
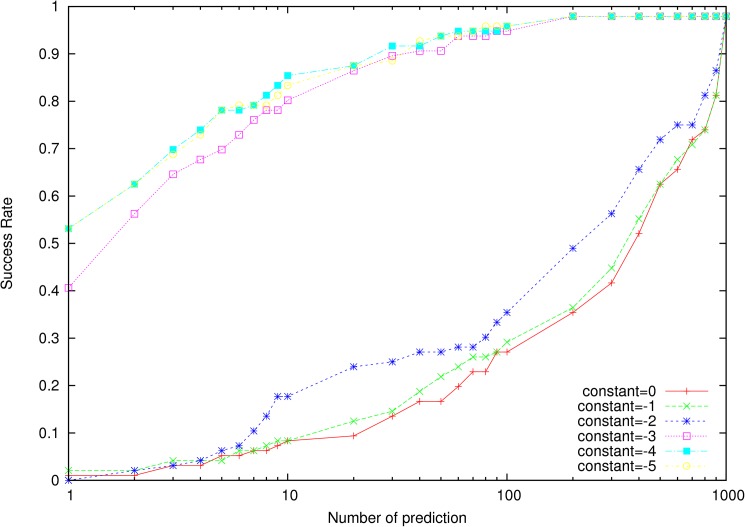
Success rates of 3dRPC-Score over the training set for different values of the constant *lnP*_*v*_ in Eq (2). *P*_*v*_ is the probability of class C in the whole conformational space of nucleotide-residue pairs in ideal state. Since the nucleotide-residue pairs are clustered into 10 classes, *P*_*v*_ = 1/10 or ln(*P*_*v*_) ≈ -2.3 and so the success rate has a dramatic change between -2 and -3.

To determine the constant in Eq (2), we ranked the training set I by using 3dRPC-Score with different values of the constant (Fig 1). It was found that when the constant is larger than -3 the success rate is very low but when the constant is less or equal to -3 the success rate becomes very high. The reason for this dramatic change will be discussed in the “Discussions” section. The scoring function gets the best performance when the constant is set as -4. Therefore, the constant was set as -4 in this work.

### Scoring RNA-protein complex

To evaluate the 3D structures of a target RNA-protein complex, the nucleotide-residue pairs are extracted from the complex and their all-atom RMSDs in relative to the standard pairs are calculated, respectively. The energy of a pair is set as that of the standard pair that has the minimum RMSD value in relative to it. The energy of the target complex is the sum of the energy of all the pairs.

$$\text{E}=\sum_{complex}E_{ij}\left(C\right)$$ (3)

## Results

To test the performance or success rate of 3dRPC-Score, we benchmark it on three unbound docking decoy datasets. To compare the performance with other scoring functions, we also give the results of DECK-RP[15] and ITScore-PR [18] since these two scoring functions have compared with other existing scoring functions and performed better than the latter. Here it is called a success prediction if the score function can pick out a near-native structure. A near-native structure is defined as a decoy structure who has a ligand RMSD (LRMSD) less than 10 Å from the native structure. Here RNAs in RNA-protein complexes are considered as ligands.

### Test on the benchmark constructed by Huang and Zou using RPDOCK

**Fig 2:**
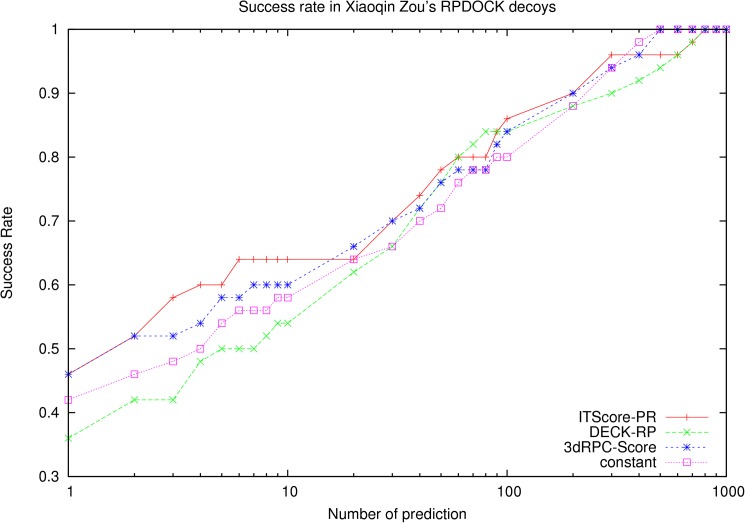
Success rates of three scoring functions on the benchmark constructed by Huang and Zou [18, 28] using RPDOCK[15].

The RNA-protein docking benchmark constructed by Huang and Zou using RPDOCK includes 72 complexes [18, 28]. The decoy sets without near-native decoys are removed, and 50 decoy sets are left. The performance of 3dRPC-Score, ITScore-PR and DECK-RP are showed in Fig 2. For the top 1 prediction, the success rates of ITScore-PR and 3dRPC-Score reach 46% and DECK-RP is 36%. Between top2 and top10 predictions the performance of ITScore-PR is slightly better than 3dRPC-Score while that of 3dRPC-Score is better than DECK-RP. For example, for top 10 predictions, the success rates of 3dRPC-Score, ITScore-PR and DECK-RP are 60%, 64% and 54%, respectively. After top 10, the performances of 3dRPC-Score and ITScore-PR are similar and are slightly better than DECK-RP. In general, on this benchmark 3dRPC-Score and ITScore-PR perform similarly and better than DECK-RP.

The success rates in three levels of difficulties (easy, medium and difficult cases) are shown in S1 Fig. For easy cases, the performances of 3dRPC-Score and ITScore-PR are similar and are better than DECK-RP; For medium cases, DECK-RP is better than 3dRPC-Score and ITScore-PR for top 2 predictions and ITScore-PR is better than 3dRPC-Score and DECK-RP when the number of prediction is larger than 3. For difficult cases, all the three scoring functions failed.

**Fig 3:**
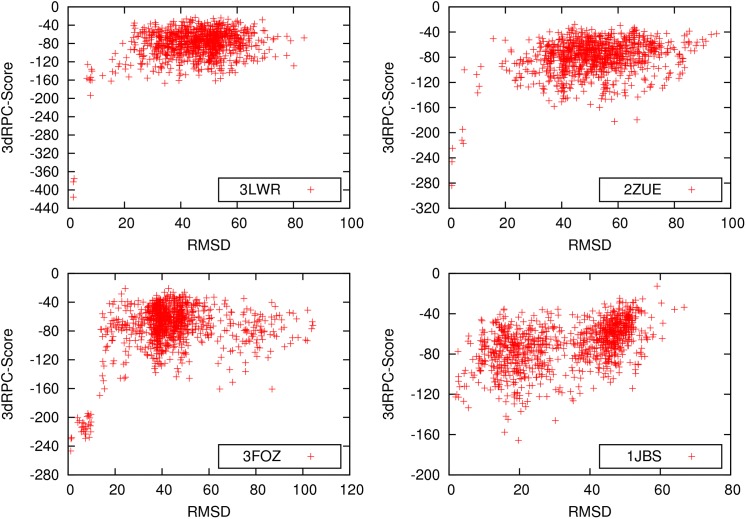
Score-LRMSD plots of the decoys of selected RNA-protein complexes.

Fig 3 shows 3dRPC-Score-LRMSD plots of the decoys of selected RNA-protein complexes. Among these four RNA-protein complexes, 3LWR, 2ZUE and 3FOZ are the cases that 3dRPC-Score successfully pick out the near-native structures while 1JBS is not. Like the other Scoring function [12, 18], the score-LRMSD plots show funnel-like shapes in the range of LRMSD < 20 Å for the first three complexes. It is interesting to note that in these three cases the near-native structures have much lower scores or “energies” than non-native structures. For the last complex (1JBS) the near-native structures do not have the lowest scores and so 3dRPC-Score failed to pick them out.

### Test on the benchmark constructed by Huang and Zou using ZDOCK[18]

**Fig 4:**
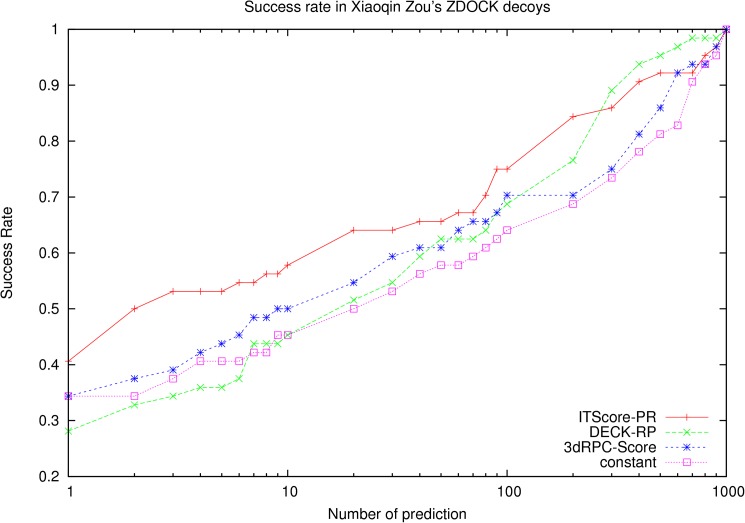
Success rates of three scoring functions on the benchmark constructed by Huang and Zou [28] using ZDOCK[30].

In this test set, we also removed the decoy sets without near-native structures, and 64 decoy sets were remained. The success rates of 3dRPC-Score, ITScore-PR and DECK-RP on this test set are showed in Fig 4. In this test set ITScore-PR performs at least 5% better than 3dRPC-Score and 3dRPC-Score better than DECK-RP. For examples, for top 1 prediction, the success rate of ITScore-PR is 40.6%, 3dRPC-Score is 34.4% and DECK-RP is 28.1%. For top 10 predictions, the success rate of ITScore-PR is 57.8%, 3dRPC-Score is 50%, and DECK-RP is 45.3%.

The success rates in three levels of difficulties (easy, medium and difficult cases) in this test set are shown in S2 Fig. For easy cases, the performance of ITScore-PR is better than 3dRPC-Score and DECK-RP; For medium cases, ITScore-PR is better than 3dRPC-Score and DECK-RP when the number of prediction is less than 30. For difficult cases, all the three scoring functions failed for small number of prediction.

**Fig 5:**
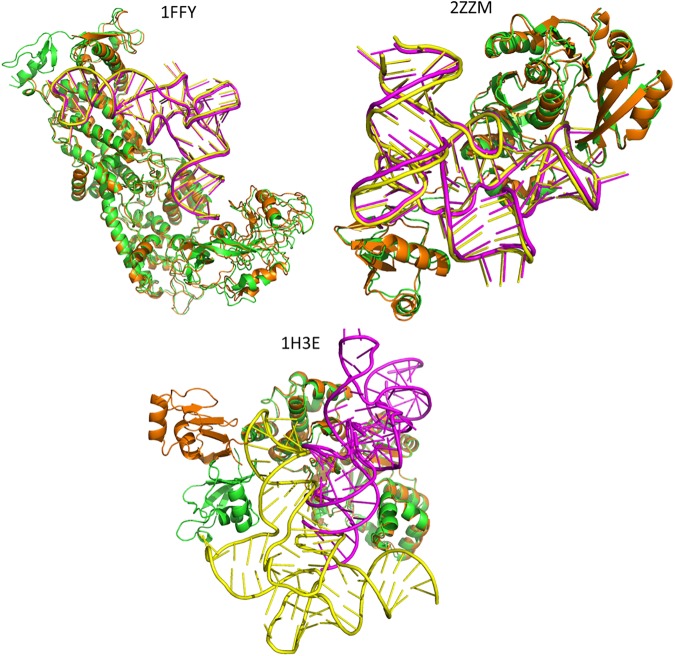
The top 1 predictions of three RNA-protein complexes comparing with their native structures by using 3dRPC-Score. The RNA and protein in native structure is in yellow and green, respectively. The RNAs and proteins in top 1 predictions are in magenta and orange, respectively. The protein RMSDs between bound and unbound states of 1FFY, 2ZZM and 1H3E are 1.367Å, 1.857 Å and 9.444Å, respectively. For 1FFY and 2ZZM, their protein conformations are similar in bound and unbound states and 3dRPC gets the near-native structure in top 1 prediction for them. For 1H3E, the bound and unbound conformations of the protein have a large change and 3dRPC-Score fails to get the near-native structure in top 1 prediction.

Fig 5 shows the top 1 predictions of three RNA-protein complexes comparing with their native structures by using 3dRPC-Score. Among them, 1FFY is “easy” case, 2ZZM “medium” and 1H3E “difficult”. For the first two (1FFY and 2ZZM) complexes the top 1 predictions completely agree with the native structures. For the third complex, the top 1 predicton is not the near-native structure because the larger conformational change of the protein.

### Test on the benchmark provided by Perez Cano et al and created using RPDOCK

**Fig 6:**
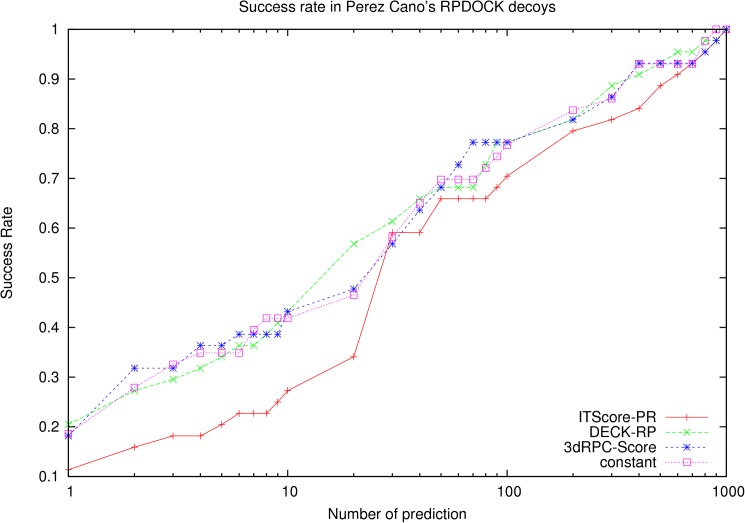
Success rates of three scoring functions on the benchmark provided by Perez Cano et al [29]and created using RPDOCK[15].

This test set includes 64 RNA-protein complexes provided by Perez Cano et al[29], and the decoy structures were generated by RPDOCK [15]. There are 43 decoy sets that include near-native structures and are used to test 3dRPC-Score. The results of 3dRPC-Score, ITScore-PR and DECK-RP are showed in Fig 6. The success rates of 3dRPC-Score and DECK-RP are close to each other and are better than ITScore-PR when the number of prediction is not larger than 20. In particular, for top 1 prediction, the success rate of DECK-RP is 20.9%, 3dRPC-Score is 18.6% and ITScore-PR is 11.6%; For top 10 predictions, the performance of 3dRPC-Score and DECK-RP is still similar (the success rate of DECK-RP are 44.2%, and the success rate of 3dRPC-Score is 41.9%), and they are much better than ITScore-PR (27.9%); When the number of predictions is larger than 30 the performance of ITScore-PR is approaching to 3dRPC-Score and DECK-RP.

The success rates in three levels of difficulties (easy, medium and difficult cases) in this test set are shown in S3 Fig. For easy cases, the performances of 3dRPC-Score and DECK-RP are better than ITScore-PR; For medium and difficult cases, the performances of the three scoring functions have no much differences.

## Discussions

As shown above, 3dRPC-Score has a consistent performance in different test sets. On Perez Cano’s test set the performance of 3dRPC-Score is similar to DECK-RP but better than ITScore-PR, on Zou’s test set constructed by RPDOCK it is similar to ITScore-PR but better than DECK-RP and on Zou’s test set constructed by ZDOCK it is worse than ITScore-PR but better than DECK-RP. We also benchmarked the three scoring functions by defining a near-native structure as a decoy structure who has a LRMSD less than 6 Å from the native structure and the results do not have significant changes (see S4 Fig).

**Fig 7:**
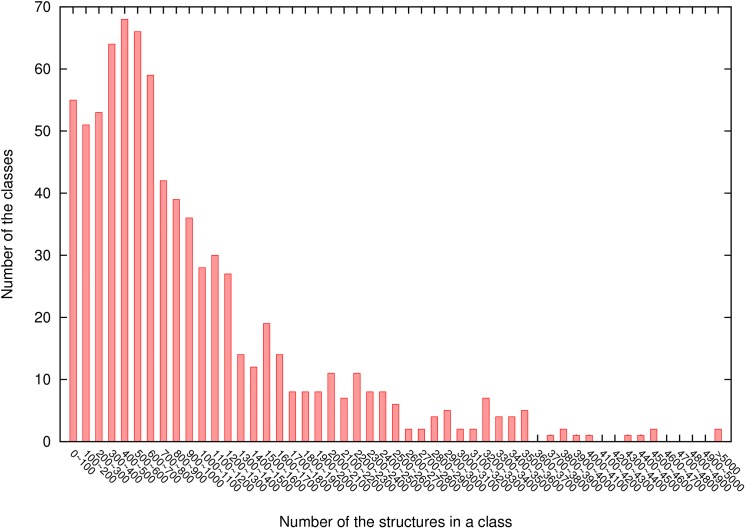
The distribution of number of conformations in each of 800 classes of nucleotide-residue pairs.

3dRPC-Score is a statistical potential that depends on the conformations of nucleotide-residue pairs, i.e., similar pair conformations are assumed to have the same energy. As shown above, we clustered the conformations of each type of nucleotide-residue pair into 10 classes by using the k-means clustering method based on their relative RMSDs. The reason of using 10 classes is to guarantee that each class has enough pair conformations for statistics. In particular we require that the numbers of pair conformations in most classes are larger than 100. In fact, only 54 out of 800 classes have conformations less than 100 when the conformations of each type of nucleotide-residue pair are clustered into 10 classes (Fig 7).

**Fig 8:**
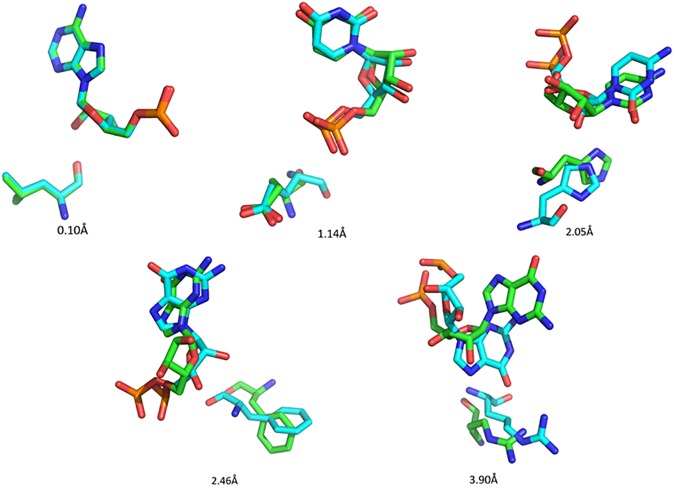
Similarity of two conformations of a nucleotide-residue pair with different relative RMSD values.

**Fig 9:**
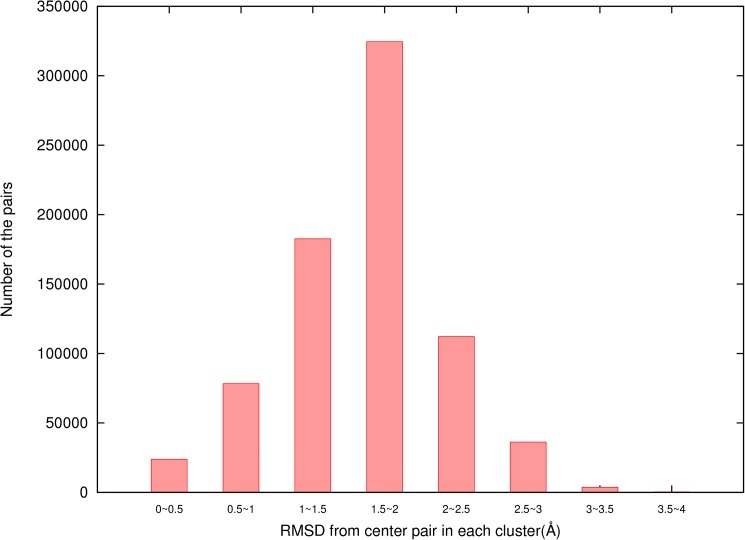
The distribution of RMSDs of nucleotide-residue conformations in relative to their center conformations of 800 classes.

Furthermore, we found that the conformations in each of 800 classes were similar. To see this, it is noted that two conformations of a nucleotide-residue pair usually are similar if their relative RMSD is less than 3 Å (Fig 8). We performed a statistics of the distribution of the largest RMSD values of the pair conformations of 800 classes in relative to their center conformations and found that the distribution was as following: 6 classes have the largest RMSD between 0Å and 1Å, 33 classes between 1Å and 2Å, 593 classes between 2Å and 3Å, and 168 classes between 3Å and 4Å. This shows that in most classes the largest RMSDs are less than 3 Å. Of course, there are also about 3/16 classes in which the largest RMSDs are between 3Å and 4Å. However, these largest-RMSD pair conformations are only small portion of each class. Fig 9 gives the distribution of RMSDs of nucleotide-residue conformations in relative to their center conformations of 800 classes. It shows that only a very small portion of the conformations have RMSD larger than 3 Å. Therefore, the conformations of the pairs in each of the 800 classes can be considered to be similar to their center conformations.

**Fig 10:**
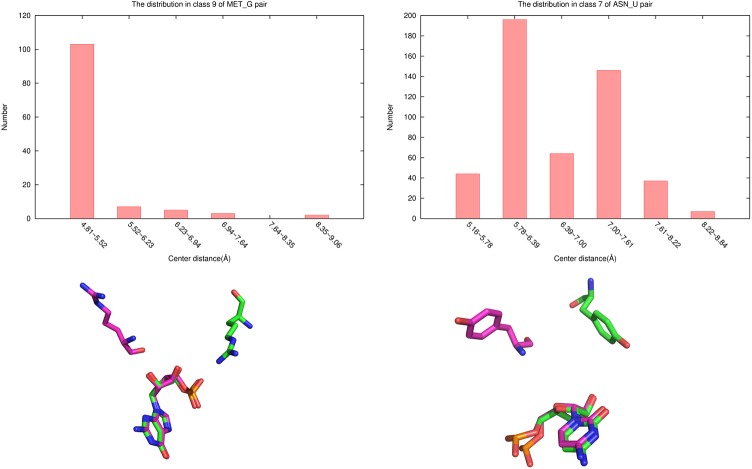
Distribution of pair conformations. Upper: the distribution of the relative distances between the nucleotide and residue in class 9 of MET-G pair (left) and class 7 from ASN-U pair; Lower: comparison of two center conformations of ARG-G (left) and TYR-C (right) pairs whose nucleotide and residue have almost the same distances but their orientations are completely different.

It is noted that in DECK-RP and ITScore-RP the energy of a nucleotide-residue pair only depends on the distance between the nucleotide and residue but not their relative orientation while in 3dRPC-Score the energy of a nucleotide-residue pair depends on the conformation of the pair. Since the conformations of each type of nucleotide-residue pair are clustered into 10 classes according to relative RMSDs, in each class the distance and orientation between the nucleotide and residue are not distributed arbitrarily but in a limited range (also see Fig 9). For examples, the relative distances of the nucleotide and residue in a class may have one or two peaks (Fig 10, upper); Furthermore, the nucleotide and residue of a pair may have different orientations in two of the 10 classes. This can be seen clearly by comparing the center conformations of two classes of a nucleotide-residue pair whose nucleotide and residue have almost the same distances but their orientations are completely different (Fig 10, lower). Therefore, 3dRPC-Score is different from the distance-dependent potential DECK_RP and ITScore-PR in that it considers both distance and orientation between the subunits of a nucleotide-residue pair.

In constructing the statistical potentials of nucleotide-residue pairs (Eq (1)), only one parameter is needed to be determined through training, i.e. *P*_*v*_ or *lnP*_*v*_ in Eq (2). Since we classified the nucleotide-residue pairs of each type into 10 classes, theoretically we should set *lnP*_*v*_ as ln(1/10) = -ln10 (about -2.3). It is interesting to note from Fig 1 that the success rate indeed has a sharp transition when the parameter changes from -2 to -3. However, since the nucleotide-residue pair information of 3dRPC-Score is from available native RNA-protein complexes in PDB, the structures of the nucleotide-residue pairs cannot cover the whole conformational space. Therefore, it may not get the best performance by using the theoretical value directly for the parameter. It is better to set the parameter through training in a bound-docking dataset. We indeed found that the best performance is achieved when *lnP*_*v*_ = −4, which is not far from -2.3.

It is noted that the constant in Eq (2) is in fact a contact potential, i.e., the potential only depends on contact number but not on contact types and conformations. It's interesting to use only the "constant" part of 3dRPC-Score to do the benchmarks. The results on the three test sets are shown in Fig 2, Fig 4 and Fig 6, respectively. It is clear that the “constant” potential performs very differently on the three test sets. On Perez Cano’s test set, its performance is similar to 3dRPC-Score and DECK-RP; On Zou’s test set constructed by RPDOCK and ZDOCK, its performance is worse than 3dRPC-Score and ITScore-PR but better than DECK-RP, at least for top 10 predictions. Therefore, the “constant” part of 3dRPC-Score plays an important role but does not dominate the results in general.

Finally, to show the advantage of 3dRPC-Score in contrast to ITScore-PR and DECK-RP, it needs to note that the three scoring functions are constructed by using different ways. 3dRPC-Score and DECK-RP were constructed by using different variables as shown above but the similar way, using a statistical potential based on Boltzmann’s formula in relative to a reference state that were trained to make the scoring functions have the best (but may not necessarily the most accurate) performance on the training set. ITScore-PR used the similar statistical potential based on Boltzmann’s formula but avoided to use the reference state. Instead it used an iterative method to make the atom-pair distribution functions approach to those of the native structures in the training set. Therefore, it is expected that ITScore-PR is good at picking out the near-native structures that are close to their native states (the near-native structures of higher quality). This can be seen from S5 Fig, which are the RMSD distributions of top 1 predictions of the three scoring functions in the three test sets. This may explain why that the success rates of ITScore-PR is much lower on Perez Cano’s test set because the near-native structures in Perez Cano’s test sets distributes much more toward to the larger RMSD end (10Å) (the end of lower quality) than those of two Xiaoqin Zou’s test sets (S6 Fig). This difference in RMSD distributions may be due to that the average contact number (or interface surface) per target in Perez Cano’s test set is lower than that in Zou’s test set (S7 Fig). In this case the performances of 3dRPC-Score and DECK-RP are almost the same as the constant potential. The reason may be that in this case most of the near-native structures have larger RMSDs in relative to the native states or looser contacts and so the energies of nucleotide-residue pairs are not very sensitive to their types and conformations and the constant part of 3dRPC-Score or the similar part (reference state) of DECK-RP dominates. This suggests that ITScore-PR is good for the decoys sets with near-native structures of higher quality while 3dRPC-Score and DECK-RP is good for the decoys sets with near-native structures of lower quality. Furthermore, since the conformation of a nucleotide-residue pair is more accurate than the distance between the pair partners in defining its energy, 3dRPC-Score should perform better than DECK-RP in general. This is clearly shown by Fig 2, Fig 4 and Fig 5 or S5 Fig.

## Conclusion

In summary, we constructed a novel pair-conformation-dependent scoring function 3dRPC-Score for evaluating 3D RNA-protein complex structures. Using conformations of nucleotide-residue pairs, the statistical potential takes account of both distance and orientation between the residue and nucleotide of a pair and this is more reasonable for constructing the statistical potential. 3dRPC-Score was tested in three different unbound docking decoy sets, and compared with DECK-RP and ITScore-PR. The performances of three scoring functions are different in different decoy sets. In particular, our results show that ITScore-PR is good for the decoys sets with near-native structures of higher quality while 3dRPC-Score is good for the decoys sets with near-native structures of lower quality. This suggests that combining the ranked results of these two scoring functions may give more accurate predictions. Furthermore, in the three test sets 3dRPC-Score has a consistent performance. These results indeed indicate the importance of considering both distance and orientation between the residue and nucleotide of a nucleotide-residue pair for statistical potentials.

## Availability

3dRPC-Score is included in 3dRPC and it can be downloaded at http://biophy.hust.edu.cn/3dRPC.html.

## Supporting information

S1 Fig The success rates for easy, medium and difficult targets in Zou’s benchmark by RPDOCK. (PDF)

S2 Fig The success rates for easy, medium and difficult targets in Zou’s benchmark by ZDOCK. (PDF)

S3 Fig The success rates for easy, medium and difficult targets in Perez Cano’s benchmark by RPDOCK. (PDF)

S4 Fig The success rate in three testing sets, when the near-native cutoff is RMSD = 6Å. (PDF)

S5 Fig The number of near-native structures within a L-RMSD value for top 1 predictions of three scoring functions in three test sets. (PDF)

S6 Fig Fraction of the number of near-native structures within a L-RMSD value for three test sets. (PDF)

S7 Fig The distribution of Average contact number per target in two test sets. The inserted plot is the total number.
(PDF)

S1 Table Training set I. The structures are used to train the parameter in statistical potential.
(PDF)

S2 Table Testing set provided by Zou. (PDF)

S3 Table Testing set provided by Perez Cano et al, which remove the same structures in training sets. (PDF)

S4 Table The selected X-ray crystal structures of ribosomes from PDB for extracting the conformations of residue-nucleotide pairs. (PDF)
