# Supplementary figures and images for: A pair-conformation-dependent scoring function for evaluating 3D RNA-protein complex structures
Source: PLoS One. 2017 Mar 30;12(3):e0174662. doi: 10.1371/journal.pone.0174662 (PMC5373608; doi:10.1371/journal.pone.0174662)

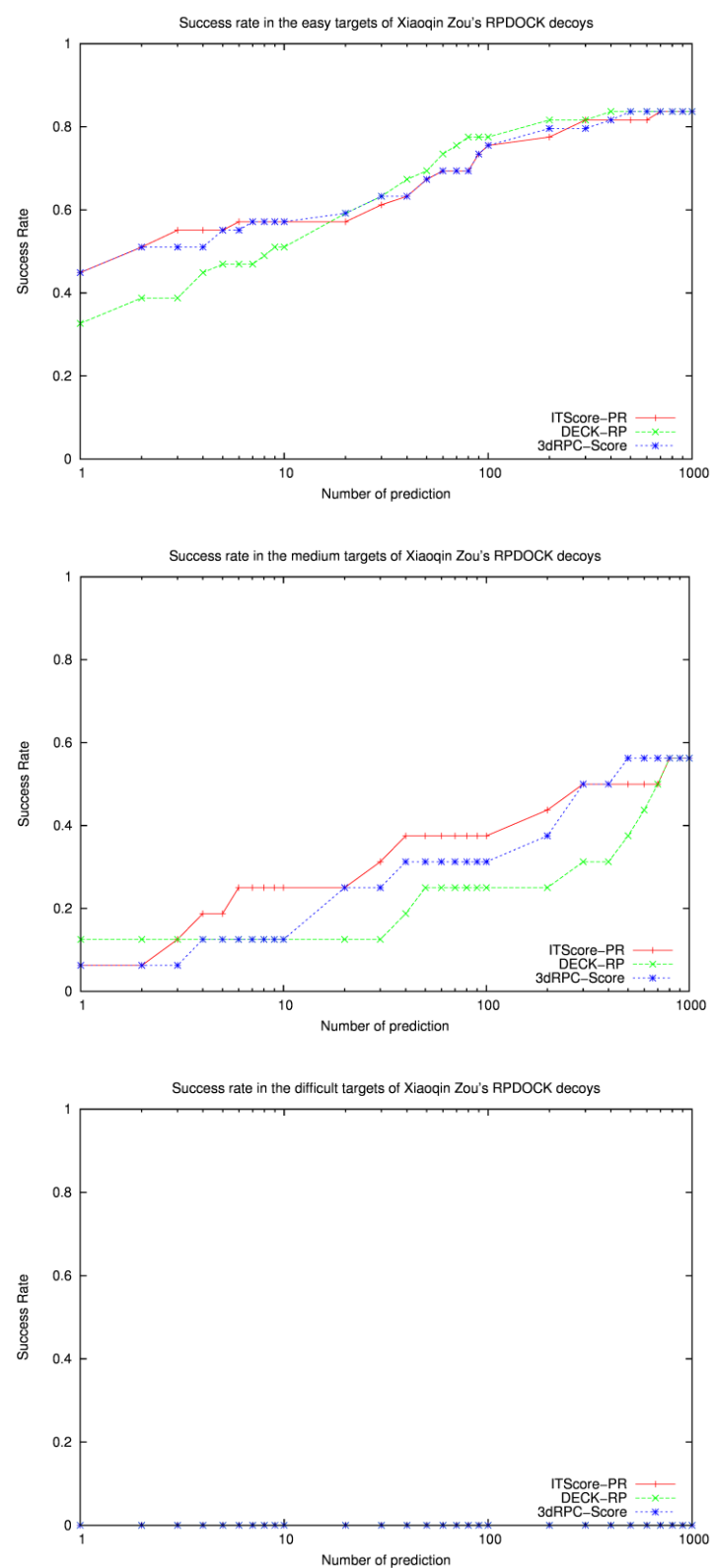

S1 Fig. The success rates for easy, medium and difficult targets in Zou's benchmark by RPDOCK.

Supplement: S1 Fig — (PDF) [file pone.0174662.s001.pdf]

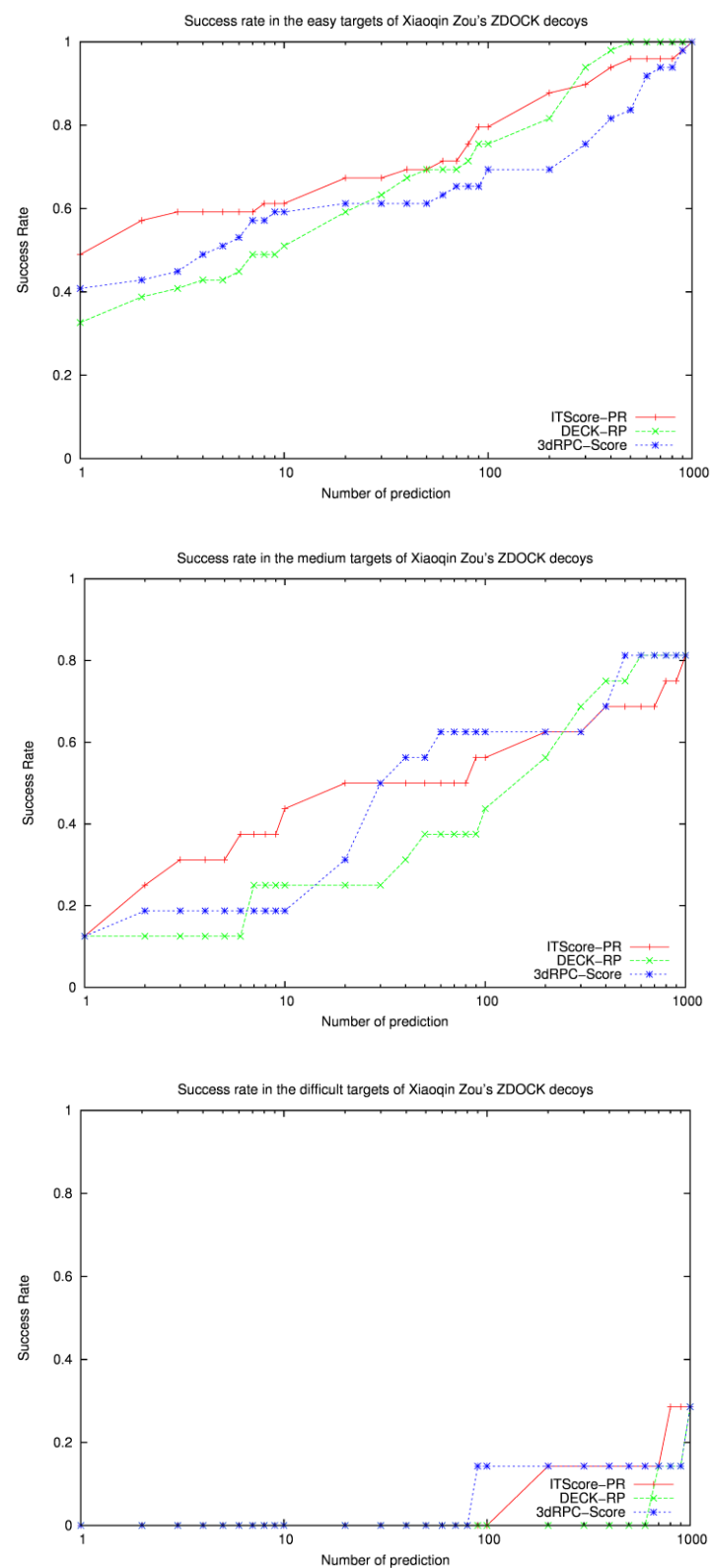

S2 Fig. The success rates for easy, medium and difficult targets in Zou's benchmark by ZDOCK.

Supplement: S2 Fig — (PDF) [file pone.0174662.s002.pdf]

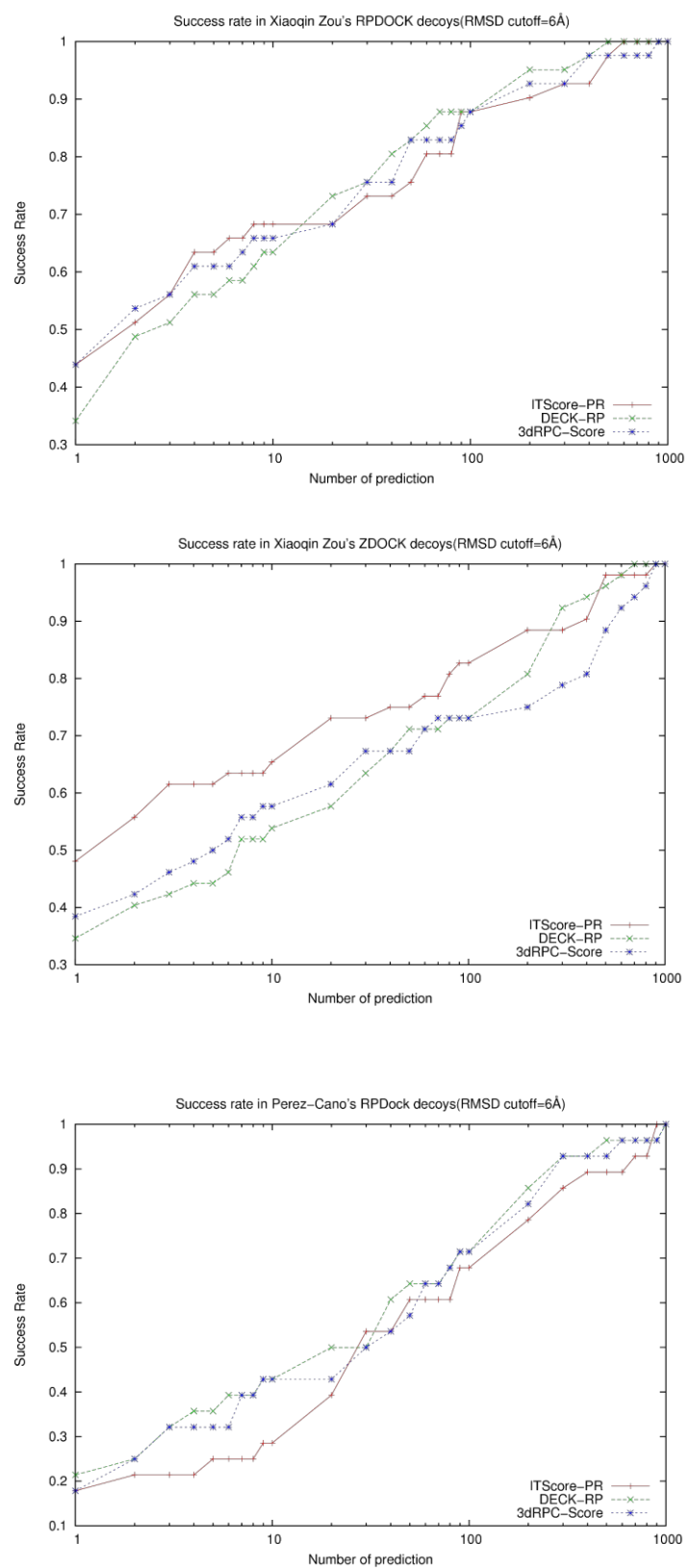

S4 Fig. The success rate in three testing sets, when the near-native cutoff is RMSD=6Å.

Supplement: S4 Fig — (PDF) [file pone.0174662.s004.pdf]

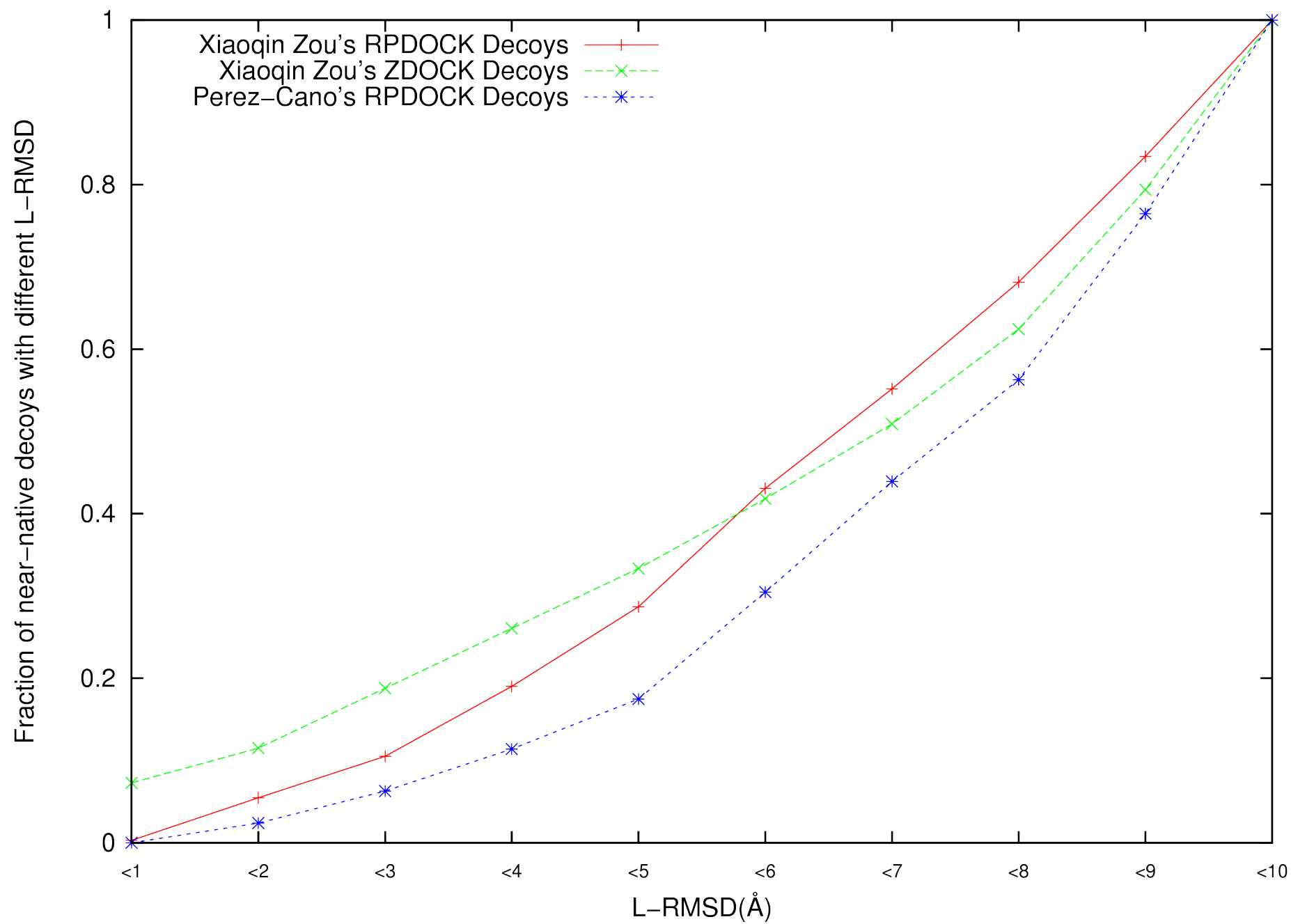

Supplement: S6 Fig — (PDF) [file pone.0174662.s006.pdf]

Average of contact in different datasets

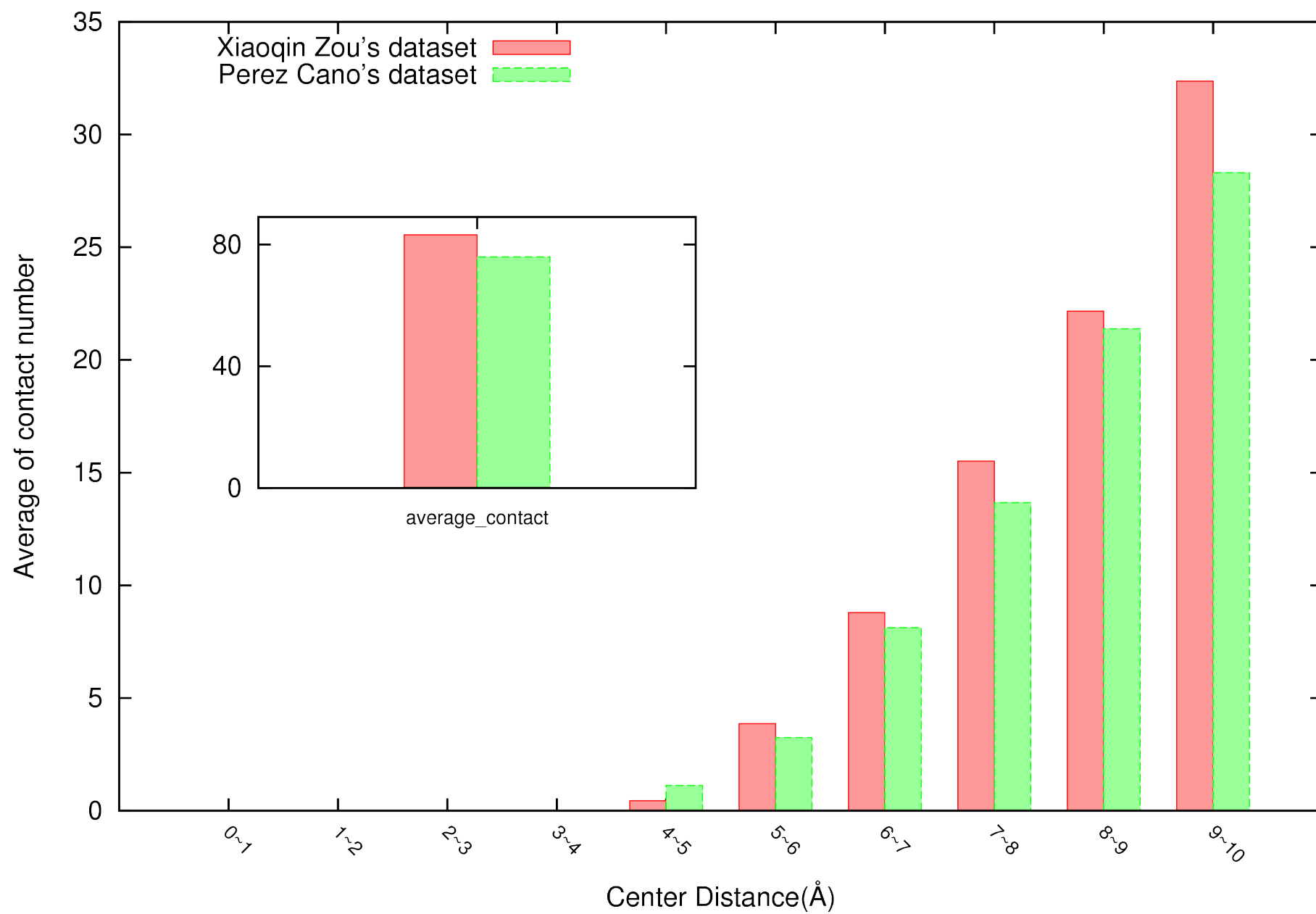

Supplement: S7 Fig — The inserted plot is the total number. (PDF) [file pone.0174662.s007.pdf]
