# Supplementary material for: A pair-conformation-dependent scoring function for evaluating 3D RNA-protein complex structures
Source: PLoS One. 2017 Mar 30;12(3):e0174662. doi: 10.1371/journal.pone.0174662 (PMC5373608; doi:10.1371/journal.pone.0174662)
Supplement: S5 Fig — (PDF) [file pone.0174662.s005.pdf]

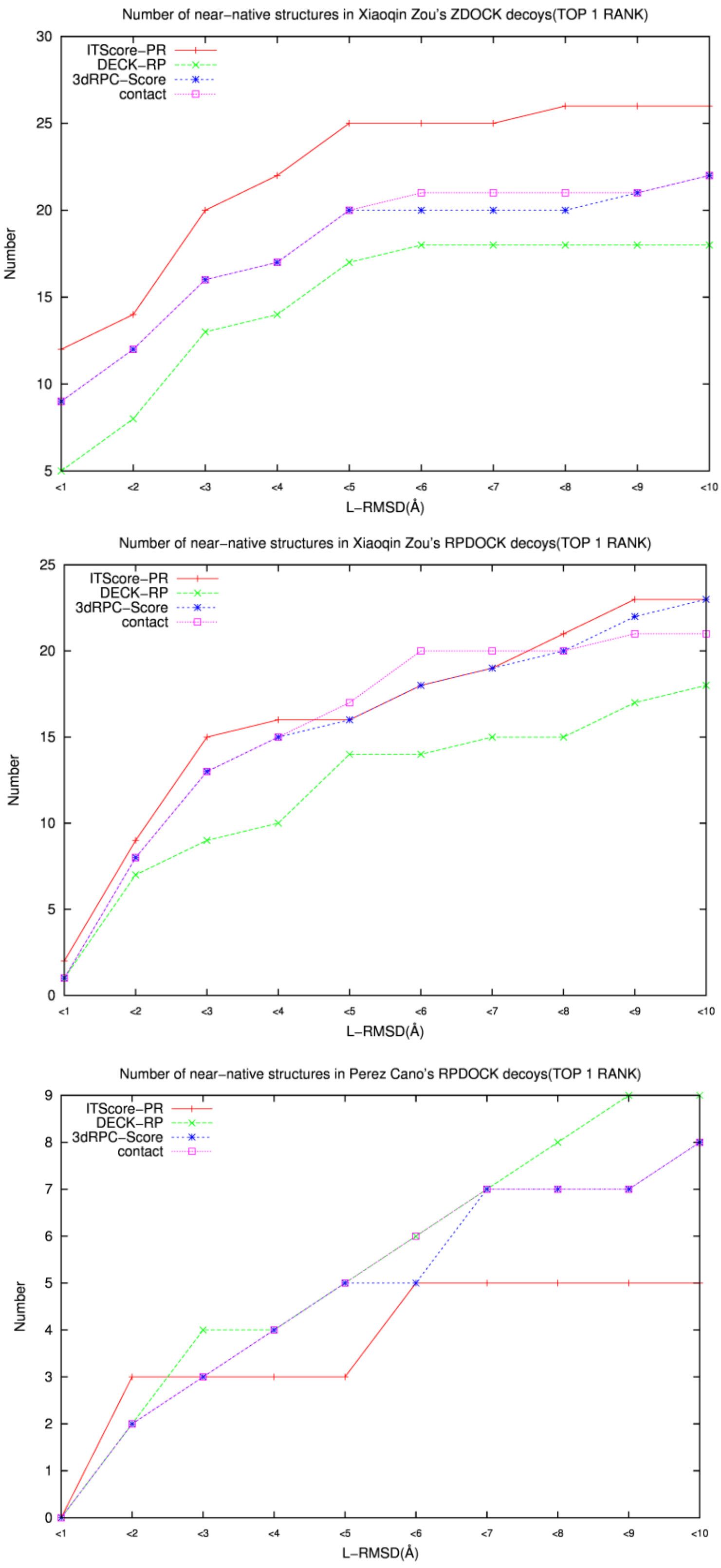

S5 Fig. The number of near-native structures within a L-RMSD value for top 1 predictions of three scoring functions in three test sets.
