# Supplementary material for: A pair-conformation-dependent scoring function for evaluating 3D RNA-protein complex structures
Source: PLoS One. 2017 Mar 30;12(3):e0174662. doi: 10.1371/journal.pone.0174662 (PMC5373608; doi:10.1371/journal.pone.0174662)
Supplement: S1 Table — The structures are used to train the parameter in statistical potential. (PDF) [file pone.0174662.s008.pdf]

S1 Table. Training set I. The structures are used to train the parameter in statistical potential.

PDBID	PROTEIN CHAIN	RNA CHAIN	PDBID	PROTEIN CHAIN	RNA CHAIN
**1A9N**	AB	Q	**3FTE**	A	CD
**1AV6**	A	B	**3G0H**	A	E
**1CVJ**	A	M	**3GIB**	ABC	H
**1CX0**	A	B	**3I5X**	A	B
**1DI2**	AB	CD	**3IAB**	AB	R
**1DUL**	A	B	**3ICE**	ABCDEF	G
**1FEU**	A	BC	**3IEV**	A	D
**1H2C**	A	R	**3K5Q**	A	B
**1L9A**	A	B	**3KS8**	AB	EF
**1N35**	A	BC	**3L25**	ABDE	CF
**1YYK**	AB	CDEF	**3MDG**	AB	C
**1RLG**	A	C	**3MQK**	A	DE
**1SI3**	A	B	**3NMR**	A	B
**1TFW**	A	GJ	**3NMU**	AG	DI
**1VFG**	A	C	**3O3I**	X	A
**1YTY**	AB	CD	**3OIJ**	AB	C
**1YYW**	A	EFGH	**3OL6**	A	BC
**1ZBH**	AD	F	**3Q0N**	A	C
**2ANN**	A	B	**3QJJ**	B	R
**2AZX**	A	C	**3R2C**	A	R
**2B3J**	AB	E	**3RC8**	A	E
**2DB3**	A	E	**3RW6**	A	H
**2DR5**	A	B	**3SIU**	AB	C
**2EZ6**	AB	CD	**3SN2**	A	B
**2F8S**	A	CD	**3SNP**	A	C
**2GXB**	A	EF	**3T5N**	A	C
**2HVY**	ABCD	E	**3TRZ**	A	U
**2I82**	A	E	**3V7E**	A	C
**2JLU**	A	C	**4E78**	A	PT
**2PJP**	A	B	**4ERD**	AB	CD
**2VNU**	D	B	**1A1T**	A	B
**2XD0**	Y	V	**1AUD**	A	B
**2XLI**	A	B	**1FJE**	B	A
**2XS2**	A	B	**1K1G**	A	B
**2XZO**	A	D	**1L1C**	AB	C

**2Y8W**	A	B	**1RGO**	A	D
**2YKG**	A	CD	**1RKJ**	A	B
**2ZI0**	A	CD	**2CJK**	A	B
**2ZZN**	A	C	**2FY1**	A	B
**3A6P**	A	DE	**2JPP**	AB	C
**3ADB**	A	C	**2KFY**	A	B
**3AEV**	AB	C	**2KH9**	A	B
**3HSB**	ABCDEF	X	**2L3C**	A	B
**3AMT**	A	B	**2LEB**	A	B
**3AVU**	A	TG	**2LI8**	A	B
**3AVY**	A	GT	**2RQC**	A	B
**3DH3**	B	F	**2XFM**	A	B
**3EQT**	AB	CD	**2YH1**	A	B
