# Supplementary material for: A pair-conformation-dependent scoring function for evaluating 3D RNA-protein complex structures
Source: PLoS One. 2017 Mar 30;12(3):e0174662. doi: 10.1371/journal.pone.0174662 (PMC5373608; doi:10.1371/journal.pone.0174662)
Supplement: S2 Table — (PDF) [file pone.0174662.s009.pdf]

S2 Table. Testing set provided by Zou

	COMPLEX		PROTEIN		RNA	
**PDBID**	PROTEIN CHAIN	RNA CHAIN	PDBID	PROTEIN CHAIN	PDBID	RNA CHAIN
			**Easy(49)**			
**1C0A**	A	B	1IL2	A	1EFW	C
**1DFU**	P	MN	3OFQ	V	1FEU	CB
**1E8O**	CD	E	1E8O	AB	1RY1	E
**1F7Y**	A	B	2VQE	O	1DK1	B
**1FFY**	A	T	1QU3	A	1QU2	T
**1G1X**	FH	IJ	2VQE	FR	1G1X	DE
**1GAX**	B	D	1GAX	A	1IVS	C
**1H4S**	AB	T	1HC7	AB	1H4Q	T
**1HQ1**	A	B	3LQX	A	1DUL	B
**1J1U**	A	B	1U7D	A	1J1U	B
**1JBS**	A	C	1JBR	A	1JBT	C
**1JID**	A	B	3KTV	B	1L1W	A
**1K8W**	A	B	1R3F	A	1ZL3	B
**1KOG**	CD	K	1EVL	AB	1KOG	I
**1LNG**	A	B	3NDB	A	2V3C	M
**1MMS**	A	C	2JQ7	A	1OLN	C
**1N78**	B	D	1J09	A	2DXI	C
**1Q2R**	C	F	1R5Y	A	1Q2S	E
**1QTQ**	A	B	1GTR	A	1QRS	B
**1R3E**	A	CDE	1ZE2	A	1R3E	CDE
**1S03**	H	A	3OFO	H	1S03	B
**1SJ3**	P	R	1M5O	C	1VC7	B
**1T0K**	B	CD	3O58	Z	1T0K	CD
**1YVP**	B	EF	1YVR	A	1YVP	CD
**2AKE**	A	B	2DR2	A	2AZX	C
**2ANR**	A	B	2ANR	A	2ANN	B
**2AZ0**	AB	CD	2B9Z	AB	2AZ2	CD
**2BH2**	A	C	1UWV	A	2BH2	D
**2CSX**	B	D	2CSX	A	2CT8	C
**2CZJ**	E	F	1WJX	A	2CZJ	B
**2DU3**	A	D	2DU5	A	2DU4	C
**2FK6**	A	R	1Y44	A	2FK6	R
**2GJW**	AB	EFH	1R0V	AB	2GJW	EFH
**2QUX**	DE	F	2QUD	AB	2QUX	C
**2RFK**	A	DE	3LWR	A	3HJY	CD

**2XDB**	A	G	2XD0	A	2XDB	G
**2ZM5**	A	C	3FOZ	A	2ZXU	C
**2ZNI**	AB	C	2ZNJ	AB	2ZNI	D
**2ZUE**	A	B	2ZUE	A	2ZUF	B
**3CIY**	A	CD	3CIG	A	3CIY	CD
**3DD2**	H	B	1GJ5	H	3DD2	B
**3EPH**	A	E	3EPK	A	3EPJ	E
**3FOZ**	A	C	2ZXU	A	2ZM5	C
**3HHZ**	O	R	3PTX	A	2GIC	R
**3LRR**	A	CD	3LRN	A	3LRR	CD
**3LWR**	ABC	DE	3LWP	ABC	3HJW	DE
**3MOJ**	B	A	2G0C	A	3MOJ	A
**3OL9**	M	NO	3OL6	A	3OLB	BC
**3OVB**	A	C	3OV7	A	3OUY	C
			**Medium(16)**			
**1F7U**	A	B	1BS2	A	1F7V	B
**1IL2**	A	C	1EQR	A	1ASY	R
**1R9F**	A	BC	1R9F	A	3CZ3	EF
**1RC7**	A	BCDE	1YYO	A	1DI2	CDEF
**1SER**	AB	T	1SES	AB	1SER	T
**1UN6**	C	E	2HGH	A	1UN6	F
**2BTE**	D	E	1H3N	A	2BYT	B
**2FMT**	A	C	1FMT	A	3CW5	A
**2NUG**	AB	CDEF	2NUF	AB	2NUG	CDEF
**2UWM**	A	C	1LVA	A	1WSU	E
**2VPL**	A	B	2OV7	A	1U63	B
**2ZKO**	AB	CD	2Z0A	AB	2ZI0	CD
**2ZZM**	A	B	2ZZN	A	2ZZM	B
**3ADD**	A	C	3ADC	A	3ADB	C
**3FTF**	A	CD	3FTD	A	3FTE	CD
**3HL2**	C	E	3HL2	A	3A3A	A
			**Difficult(7)**			
**1H3E**	A	B	1H3F	A	1H3E	B
**1OOA**	B	D	1NFK	A	2JWV	A
**1U0B**	B	A	1LI5	A	1B23	R
**2HW8**	A	B	1AD2	A	1ZHO	B
**2IPY**	A	C	2B3Y	A	2IPY	D
**2R8S**	HL	R	3IVK	AB	1HR2	A
**2V3C**	C	M	3NDB	B	1LNG	B
