# Supplementary material for: A pair-conformation-dependent scoring function for evaluating 3D RNA-protein complex structures
Source: PLoS One. 2017 Mar 30;12(3):e0174662. doi: 10.1371/journal.pone.0174662 (PMC5373608; doi:10.1371/journal.pone.0174662)
Supplement: S3 Table — (PDF) [file pone.0174662.s010.pdf]

S3 Table. Testing set provided by Perez Cano et al, which remove the same structures in training sets.

COMPLEX			PROTEIN		RNA	
**PDBID**	PROTEIN CHAIN	RNA CHAIN	PDBID	PROTEIN CHAIN	PDBID	RNA CHAIN
			**Easy(46)**			
**1C9S**	LMNOPQRSTUV	W	1QAW	ABCDEFGHIJK	1C9S	W
**1E7K**	A	C	2JNB	A	1E7K	C
**1EC6**	A	D	1DTJ	A	1EC6	D
**1G1X**	A	D	1RIS	A	1G1X	D
**1H3E**	A	B	1H3F	A	1H3E	B
**1H4S**	AB	T	1HC7	AB	1H4S	T
**1JBR**	B	D	1AQZ	A	1JBR	D
**1K8W**	A	B	1R3F	A	1K8W	B
**1KOG**	A	I	1EVL	A	1KOG	I
**1KQ2**	ABHIKM	R	1KQ1	ABHIKM	1KQ2	R
**1M5O**	C	AB	1NU4	A	1M5O	AB
**1M8V**	ABCDEFG	O	1H64	ABCDEFG	1M8V	O
**1M8W**	A	C	1M8Z	A	1M8W	C
**1N78**	A	C	1J09	A	1N78	C
**1Q2R**	A	E	1R5Y	A	1Q2R	E
**1SER**	AB	T	1SES	AB	1SER	T
**1T4L**	B	A	1T4O	A	1T4L	A
**1U63**	A	B	1I2A	A	1U63	B
**1WNE**	A	BC	1U09	A	1WNE	BC
**1WPU**	A	C	1WPV	A	1WPU	C
**1WSU**	A	E	1LVA	A	1WSU	E
**1YVP**	A	CD	1YVR	A	1YVP	CD
**2AD9**	A	B	1SJQ	A	2AD9	B
**2ADB**	A	B	1SJR	A	2ADB	B
**2ASB**	A	B	1K0R	A	2ASB	B
**2AZ0**	AB	CD	2B9Z	AB	2AZ0	CD
**2BGG**	A	PQ	1W9H	A	2BGG	PQ
**2BH2**	A	C	1UWV	A	2BH2	C
**2BTE**	A	B	1H3N	A	2BTE	B
**2BU1**	A	R	2MS2	A	2BU1	R
**2CZJ**	A	B	1WJX	A	2CZJ	B
**2ERR**	A	B	2CQ3	A	2ERR	B

**2F8K**	A	B	2D3D	A	2F8K	B
**2FMT**	A	C	1FMT	A	3CW5	A
**2GJW**	AB	EFH	1R0V	AB	2GJW	EFH
**2I91**	B	EF	1YVR	A	2I91	EF
**2IX1**	A	B	2ID0	A	2IX1	B
**2PY9**	A	E	2JZX	A	2PY9	E
**2QUX**	AB	C	2QUD	AB	2QUX	C
**2R7R**	A	X	2R7Q	A	2R7R	X
**3BO2**	A	BCDE	1NU4	A	3BO2	BCDE
**3BSB**	B	C	1M8Z	A	3BSB	C
**3BSO**	A	PT	1SH0	A	3BSO	PT
**3BSX**	A	C	1M8Z	A	3BSX	C
**3BX2**	A	C	1M8Z	A	3BX2	C
**3CIY**	A	CD	3CIG	A	3CIY	CD
			**Medium(11)**			
**1ASY**	AB	R	1EOV	AB	3TRA	A
**1DFU**	P	MN	1B75	A	364D	BC
**1EKZ**	A	B	1STU	A	1EKZ	B
**1F7U**	A	B	1BS2	A	1F7U	B
**1MFQ**	C	A	1QB2	B	1L9A	B
**1MMS**	A	C	2K3F	A	1MMS	C
**1QTQ**	A	B	1NYL	A	3KNH	Y
**1T0K**	AB	CD	1NMU	AB	1T0K	CD
**1U0B**	B	A	1LI7	A	1B23	R
**2ADC**	A	B	2EVZ	A	2ADC	B
**2GJE**	AD	RS	2GIA	AB	2GJE	RS
			**Difficult(7)**			
**1OB2**	A	B	1EFC	A	1EHZ	A
**1R3E**	A	C	1ZE1	A	1EHZ	A
**1B23**	P	R	1TUI	A	1U0B	A
**1B7F**	A	P	3SXL	A	1B7F	P
**1HVU**	AB	C	2VG5	AB	1HVU	C
**2HGH**	A	B	2J7J	A	2HGH	B
**2HW8**	A	B	1AD2	A	2HW8	B
