# Supplementary material for: A pair-conformation-dependent scoring function for evaluating 3D RNA-protein complex structures
Source: PLoS One. 2017 Mar 30;12(3):e0174662. doi: 10.1371/journal.pone.0174662 (PMC5373608; doi:10.1371/journal.pone.0174662)
Supplement: S4 Table — (PDF) [file pone.0174662.s011.pdf]

S4 Table. The selected X-ray crystal structures of ribosomes from PDB for extracting the conformations of residue-nucleotide pairs.

PDBID	PROTEIN CHAIN	RNA CHAIN
**1DK1**	A	B
**1FEU**	AD	BCEF
**1FFK**	ABCDEFGHIJKLMNOPQRSTUVWXYZ1	9
**1FJG**	BCDEFGHIJKLMNOPQRSTV	AX
**1HC8**	AB	CD
**1I6U**	AB	CD
**1JJ2**	ABCDEFGHIJKLMNOPQRSTUVWXYZ12	9
**1K8A**	CDEFGHIJKLMNOPQRSTUVWXYZ1234	AB
**1K9M**	CDEFGHIJKLMNOPQRSTUVWXYZ1234	AB
**1KD1**	CDEFGHIJKLMNOPQRSTUVWXYZ1234	AB
**1KUQ**	A	B
**1M90**	CDEFGHIJKLMNOPQRSTUVWXYZ1234	AB5
**1MJI**	AB	CD
**1MZP**	A	B
**1N32**	BCDEFGHIJKLMNOPQRSTV	AYZ
**1N8R**	CDEFGHIJKLMNOPQRSTUVWXYZ1234	AB
**1NJI**	CDEFGHIJKLMNOPQRSTUVWXYZ1234	AB
**1Q81**	CDEFGHIJKLMNOPQRSTUVWXYZ1234	AB5
**1Q82**	CDEFGHIJKLMNOPQRSTUVWXYZ1234	AB5

**1Q86**	CDEFGHIJKLMNOPQRSTUVWXYZ1234	AB56
**1QA6**	AB	CD
**1QVG**	ABCDEFGHIJKLMNOPQRSTUVWXYZ12	9345
**1S72**	ABCDEFGHIJKLMNOPQRSTUVWXYZ123	9
**1TTT**	ABC	DEF
**1VQ4**	ABCDEFGHJKLMNOPQRSTUVWXYZ123I	94
**1VQ5**	ABCDEFGHJKLMNOPQRSTUVWXYZ123I	94
**1VQ6**	ABCDEFGHJKLMNOPQRSTUVWXYZ123I	945
**1VQ7**	ABCDEFGHJKLMNOPQRSTUVWXYZ123I	94
**1VQ8**	ABCDEFGHJKLMNOPQRSTUVWXYZ123I	94
**1VQ9**	ABCDEFGHJKLMNOPQRSTUVWXYZ123I	94
**1VQK**	ABCDEFGHJKLMNOPQRSTUVWXYZ123I	94
**1VQL**	ABCDEFGHJKLMNOPQRSTUVWXYZ123I	94
**1VQM**	ABCDEFGHJKLMNOPQRSTUVWXYZ123I	94
**1VQN**	ABCDEFGHJKLMNOPQRSTUVWXYZ123I	945
**1VQO**	ABCDEFGHJKLMNOPQRSTUVWXYZ123I	94
**1VQP**	ABCDEFGHJKLMNOPQRSTUVWXYZ123I	94
**1XMQ**	BCDEFGHIJKLMNOPQRSTV	AWX
**1YHQ**	ABCDEFGHIJKLMNOPQRSTUVWXYZ123	9
**1YI2**	ABCDEFGHIJKLMNOPQRSTUVWXYZ123	9
**1YIJ**	ABCDEFGHIJKLMNOPQRSTUVWXYZ123	9
**1YIT**	1238ABCDEFGHIJKLMNOPQRSTUVWXYZ	9
**1YJ9**	ABCDEFGHIJKLMNOPQRSTUVWXYZ123	9
**1YJN**	ABCDEFGHIJKLMNOPQRSTUVWXYZ123	9
**1YJW**	1234ABCDEFGHIJKLMNOPQRSTUVWXYZ	9
**1ZHO**	ACEG	BDFH

**2HVY**	ABCD	E
**2OTJ**	ABCDEFGHJKLMNOPQRSTUVWXYZ123I	9
**2OTL**	ABCDEFGHJKLMNOPQRSTUVWXYZ123I	9
**2QA4**	ABCDEFGHIJKLMNOPQRSTUVWXYZ123	9
**2QEX**	ABCDEFGHJKLMNOPQRSTUVWXYZ123I	9
**2UUA**	BCDEFGHIJKLMNOPQRSTU	AXY
**2UUB**	BCDEFGHIJKLMNOPQRSTU	AXY
**2UXC**	BCDEFGHIJKLMNOPQRSTU	AXY
**2VQE**	BCDEFGHIJKLMNOPQRSTU	AXY
**2VQF**	BCDEFGHIJKLMNOPQRSTU	AXY
**2ZJR**	ABCDEFGHIJKLMNOPQRSTUVWZ1234	XY
**3CC2**	ABCDEFGHIJKLMNOPQRSTUVWXYZ123	9
**3CC4**	ABCDEFGHIJKLMNOPQRSTUVWXYZ123	9
**3CC7**	ABCDEFGHIJKLMNOPQRSTUVWXYZ123	9
**3CCE**	ABCDEFGHIJKLMNOPQRSTUVWXYZ123	9
**3CCJ**	ABCDEFGHIJKLMNOPQRSTUVWXYZ123	9
**3CCL**	ABCDEFGHIJKLMNOPQRSTUVWXYZ123	9
**3CCM**	ABCDEFGHIJKLMNOPQRSTUVWXYZ123	9
**3CCQ**	ABCDEFGHIJKLMNOPQRSTUVWXYZ123	9
**3CCR**	ABCDEFGHIJKLMNOPQRSTUVWXYZ123	9
**3CCS**	ABCDEFGHIJKLMNOPQRSTUVWXYZ123	9
**3CCU**	ABCDEFGHIJKLMNOPQRSTUVWXYZ123	9
**3CCV**	ABCDEFGHIJKLMNOPQRSTUVWXYZ123	9
**3CD6**	ABCDEFGHIJKLMNOPQRSTUVWXYZ123	94
**3CMA**	ABCDEFGHIJKLMNOPQRSTUVWXYZ123	956
**3CME**	ABCDEFGHIJKLMNOPQRSTUVWXYZ123	956

**3CPW**	ABCDEFGHIJKLMNOPQRSTUVWXYZ12	94
**3CXC**	ABCDEFGHIJKLMNOPQRSTUVWXYZ12	94
**3DH3**	ABCD	EFGH
**3G6E**	ABCDEFGHIJKLMNOPQRSTUVWXYZ123	9
**3G71**	ABCDEFGHIJKLMNOPQRSTUVWXYZ123	9
**3HAX**	ACD	EF
**3HJW**	ABC	DE
**3I56**	ABCDEFGHIJKLMNOPQRSTUVWXYZ123	9
**3IEV**	A	D
**3LWO**	ABC	DE
**3LWP**	ABC	DE
**3LWQ**	ABC	DE
**3LWV**	ABC	DE
**3MQK**	ABC	DE
**3NVI**	ABCD	EF
**3OIJ**	AB	CD
**3OIN**	AB	C
**3OW2**	ABCDEFGHIJKLMNOPQRSTUVWXYZ12	9
**3R2C**	ABJK	RS
**3R2D**	ABJK	RS
**3R9W**	A	B
**3R9X**	AB	C
**3RTJ**	AB	D
**3T1Y**	BCDEFGHIJKLMNOPQRSTV	AWX
**3U4M**	A	B
**3U56**	A	B

**3UMY**		A	B
**3V7E**		AB	CD
**4B3M**		BCDEFGHIJKLMNOPQRSTV	AWZ
**4B3R**		BCDEFGHIJKLMNOPQRSTV	AWZ
**4B3T**		BCDEFGHIJKLMNOPQRSTV	AWZ
**4QG3**		A	B
**4QVI**		A	B
**1VY4**	1vy4-pdb-bundle1.pdb	BCDEFGHIJKLMNOPQRSTU	AVWXY
	1vy4-pdb-bundle2.pdb	CDEFGHIJKLMNOPQRSTUVWXYZabcde	AB
	1vy4-pdb-bundle3.pdb	BCDEFGHIJKLMNOPQRSTU	AVWXY
	1vy4-pdb-bundle4.pdb	CDEFGHIJKLMNOPQRSTUVWXYZabcde	AB
**1VY5**	1vy5-pdb-bundle1.pdb	BCDEFGHIJKLMNOPQRSTU	AVWXY
	1vy5-pdb-bundle2.pdb	CDEFGHIJKLMNOPQRSTUVWXYZabcde	AB
	1vy5-pdb-bundle3.pdb	BCDEFGHIJKLMNOPQRSTU	AVWXY
	1vy5-pdb-bundle4.pdb	CDEFGHIJKLMNOPQRSTUVWXYZabcde	AB
**1VY6**	1vy6-pdb-bundle1.pdb	BCDEFGHIJKLMNOPQRSTU	AVWX
	1vy6-pdb-bundle2.pdb	CDEFGHIJKLMNOPQRSTUVWXYZabcde	AB
	1vy6-pdb-bundle3.pdb	BCDEFGHIJKLMNOPQRSTU	AVWX
	1vy6-pdb-bundle4.pdb	CDEFGHIJKLMNOPQRSTUVWXYZabcde	AB
**1VY7**	1vy7-pdb-bundle1.pdb	BCDEFGHIJKLMNOPQRSTU	AVWXY
	1vy7-pdb-bundle2.pdb	CDEFGHIJKLMNOPQRSTUVWXYZabcde	AB
	1vy7-pdb-bundle3.pdb	BCDEFGHIJKLMNOPQRSTU	AVWXY
	1vy7-pdb-bundle4.pdb	CDEFGHIJKLMNOPQRSTUVWXYZabcde	AB
**4LNT**	4lnt-pdb-bundle1.pdb	BCDEFGHIJKLMNOPQRSTU	AVWX
	4lnt-pdb-bundle2.pdb	CDEFGHIJKLMNOPQRSTUVWXYZabcde	AB
	4lnt-pdb-bundle3.pdb	BCDEFGHIJKLMNOPQRSTU	AVWX

	4lnt-pdb-bundle4.pdb	CDEFGHIJKLMNOPQRSTUVWXYZabcde	ABfg
**4U1U**	4u1u-pdb-bundle1.pdb	BCDEFGHIJKLMNOPQRSTU	A
	4u1u-pdb-bundle2.pdb	CDEFGHIJKLMNOPQRSTUVWXYZabcdefg	AB
	4u1u-pdb-bundle3.pdb	BCDEFGHIJKLMNOPQRSTU	A
	4u1u-pdb-bundle4.pdb	CDEFGHIJKLMNOPQRSTUVWXYZabcdef	AB
**4U1V**	4u1v-pdb-bundle1.pdb	BCDEFGHIJKLMNOPQRSTU	A
	4u1v-pdb-bundle2.pdb	CDEFGHIJKLMNOPQRSTUVWXYZabcdefg	AB
	4u1v-pdb-bundle3.pdb	BCDEFGHIJKLMNOPQRSTU	A
	4u1v-pdb-bundle4.pdb	CDEFGHIJKLMNOPQRSTUVWXYZabcdef	AB
**4U20**	4u20-pdb-bundle1.pdb	BCDEFGHIJKLMNOPQRSTU	A
	4u20-pdb-bundle2.pdb	CDEFGHIJKLMNOPQRSTUVWXYZabcdef	AB
	4u20-pdb-bundle3.pdb	BCDEFGHIJKLMNOPQRSTU	A
	4u20-pdb-bundle4.pdb	CDEFGHIJKLMNOPQRSTUVWXYZabcde	AB
**4U24**	4u24-pdb-bundle1.pdb	BCDEFGHIJKLMNOPQRSTU	A
	4u24-pdb-bundle2.pdb	CDEFGHIJKLMNOPQRSTUVWXYZabcdef	AB
	4u24-pdb-bundle3.pdb	BCDEFGHIJKLMNOPQRSTU	A
	4u24-pdb-bundle4.pdb	CDEFGHIJKLMNOPQRSTUVWXYZabcde	AB
**4U25**	4u25-pdb-bundle1.pdb	BCDEFGHIJKLMNOPQRSTU	A
	4u25-pdb-bundle2.pdb	CDEFGHIJKLMNOPQRSTUVWXYZabcdef	AB
	4u25-pdb-bundle3.pdb	BCDEFGHIJKLMNOPQRSTU	A
	4u25-pdb-bundle4.pdb	CDEFGHIJKLMNOPQRSTUVWXYZabcde	AB
**4U26**	4u26-pdb-bundle1.pdb	BCDEFGHIJKLMNOPQRSTU	A
	4u26-pdb-bundle2.pdb	CDEFGHIJKLMNOPQRSTUVWXYZabcdefg	AB
	4u26-pdb-bundle3.pdb	BCDEFGHIJKLMNOPQRSTU	A
	4u26-pdb-bundle4.pdb	CDEFGHIJKLMNOPQRSTUVWXYZabcdef	AB
**4U27**	4u27-pdb-bundle1.pdb	BCDEFGHIJKLMNOPQRSTU	A

	4u27-pdb-bundle2.pdb	CDEFGHIJKLMNOPQRSTUVWXYZabcdefg	AB
	4u27-pdb-bundle3.pdb	BCDEFGHIJKLMNOPQRSTU	A
	4u27-pdb-bundle4.pdb	CDEFGHIJKLMNOPQRSTUVWXYZabcdef	AB
	4u3m-pdb-bundle1.pdb	BCDEFGHIJKLMNOPQRSTUVWXYZabcdefghi	A
	4u3m-pdb-bundle2.pdb	DEFGHIJKLMNO	ABC
**4U3M**	4u3m-pdb-bundle3.pdb	ABCDEFGHIJKLMNOPQRSTUVWXYZabcefghijklmnopqrstuvwxyz0123	d
	4u3m-pdb-bundle4.pdb	ABCDEFGHLMNOPQRS	IJK
	4u3m-pdb-bundle5.pdb	ABCDEFGHIJKLMNOPQRSTUVWXYZabcdefghijk	
	4u3u-pdb-bundle1.pdb	BCDEFGHIJKLMNOPQRSTUVWXYZabcdefghi	A
	4u3u-pdb-bundle2.pdb	DEFGHIJKLMNO	ABC
**4U3U**	4u3u-pdb-bundle3.pdb	ABCDEFGHIJKLMNOPQRSTUVWXYZabcefghijklmnopqrstuvwxyz0123	d
	4u3u-pdb-bundle4.pdb	ABCDEFGHLMNOPQRS	IJK
	4u3u-pdb-bundle5.pdb	ABCDEFGHIJKLMNOPQRSTUVWXYZabcdefghijk	
	4u4q-pdb-bundle1.pdb	BCDEFGHIJKLMNOPQRSTUVWXYZabcdefghi	A
	4u4q-pdb-bundle2.pdb	DEFGHIJKLMNO	ABC
**4U4Q**	4u4q-pdb-bundle3.pdb	ABCDEFGHIJKLMNOPQRSTUVWXYZabcefghijklmnopqrstuvwxyz0123	d
	4u4q-pdb-bundle4.pdb	ABCDEFGHLMNOPQRS	IJK
	4u4q-pdb-bundle5.pdb	ABCDEFGHIJKLMNOPQRSTUVWXYZabcdefghijk	
	4u4r-pdb-bundle1.pdb	BCDEFGHIJKLMNOPQRSTUVWXYZabcdefghi	A
	4u4r-pdb-bundle2.pdb	DEFGHIJKLMNO	ABC
**4U4R**	4u4r-pdb-bundle3.pdb	ABCDEFGHIJKLMNOPQRSTUVWXYZabcefghijklmnopqrstuvwxyz0123	d
	4u4r-pdb-bundle4.pdb	ABCDEFGHLMNOPQRS	IJK
	4u4r-pdb-bundle5.pdb	ABCDEFGHIJKLMNOPQRSTUVWXYZabcdefghijk	
	4u4u-pdb-bundle1.pdb	BCDEFGHIJKLMNOPQRSTUVWXYZabcdefghi	A
**4U4U**	4u4u-pdb-bundle2.pdb	DEFGHIJKLMNO	ABC
	4u4u-pdb-bundle3.pdb	ABCDEFGHIJKLMNOPQRSTUVWXYZabcefghijklmnopqrstuvwxyz0123	d

	4u4u-pdb-bundle4.pdb	ABCDEFGHLMNOPQRS	IJK
	4u4u-pdb-bundle5.pdb	ABCDEFGHIJKLMNOPQRSTUVWXYZabcdefghijk	
4U52	4u52-pdb-bundle1.pdb	BCDEFGHIJKLMNOPQRSTUVWXYZabcdefghi	A
	4u52-pdb-bundle2.pdb	DEFGHIJKLMNO	ABC
	4u52-pdb-bundle3.pdb	ABCDEFGHIJKLMNOPQRSTUVWXYZabcefghijklmnopqrstuvwxyz0123	d
	4u52-pdb-bundle4.pdb	ABCDEFGHLMNOPQRS	IJK
	4u52-pdb-bundle5.pdb	ABCDEFGHIJKLMNOPQRSTUVWXYZabcdefghijk	
4V51	4v51-pdb-bundle1.pdb	BCDEFGHIJKLMNOPQRSTUZabcdefgh	AVWXY
	4v51-pdb-bundle2.pdb	CDEFGHIJKLMNOPQRSTUV	AB
	4v51-pdb-bundle3.pdb	BCDEFGHIJKLMNOPQRSTUZabcdefgh	AVWXY
	4v51-pdb-bundle4.pdb	CDEFGHIJKLMNOPQRSTUV	AB
4V67	4v67-pdb-bundle1.pdb	EFGHIJKLMNOPQRSTUVWXY	ABCD
	4v67-pdb-bundle2.pdb	CDEFGHIJKLMNOPQRSTUVWXYZabcde	AB
	4v67-pdb-bundle3.pdb	EFGHIJKLMNOPQRSTUVWXY	ABCD
	4v67-pdb-bundle4.pdb	CDEFGHIJKLMNOPQRSTUVWXYZabcde	AB
4V7L	4v7l-pdb-bundle1.pdb	BCDEFGHIJKLMNOPQRSTUZ	AVWXY
	4v7l-pdb-bundle2.pdb	CDEFGHIJKLMNOPQRSTUVWXYZabcdef	AB
	4v7l-pdb-bundle3.pdb	BCDEFGHIJKLMNOPQRSTUZ	AVWXY
	4v7l-pdb-bundle4.pdb	CDEFGHIJKLMNOPQRSTUVWXYZabcdef	AB
4V7W	4v7w-pdb-bundle1.pdb	BCDEFGHIJKLMNOPQRSTUVWXYZabcd	A
	4v7w-pdb-bundle2.pdb	CDEFGHIJKLMNOPQRSTU	AB
	4v7w-pdb-bundle3.pdb	BCDEFGHIJKLMNOPQRSTUVWXYZabcd	A
	4v7w-pdb-bundle4.pdb	CDEFGHIJKLMNOPQRSTU	AB
4V7X	4v7x-pdb-bundle1.pdb	BCDEFGHIJKLMNOPQRSTUVWXYZabcd	A
	4v7x-pdb-bundle2.pdb	CDEFGHIJKLMNOPQRSTU	AB
	4v7x-pdb-bundle3.pdb	BCDEFGHIJKLMNOPQRSTUVWXYZabcd	A

	4v7x-pdb-bundle4.pdb	CDEFGHIJKLMNOPQRSTU	AB
4V7Y	4v7y-pdb-bundle1.pdb	BCDEFGHIJKLMNOPQRSTUVWXYZabcd	A
	4v7y-pdb-bundle2.pdb	CDEFGHIJKLMNOPQRSTU	AB
	4v7y-pdb-bundle3.pdb	BCDEFGHIJKLMNOPQRSTUVWXYZabcd	A
	4v7y-pdb-bundle4.pdb	CDEFGHIJKLMNOPQRSTU	AB
4V88	4v88-pdb-bundle1.pdb	BCDEFGHIJKLMNOPQRSTUVWXYZabcdefghi	A
	4v88-pdb-bundle2.pdb	DEFGHIJKLMNO	ABC
	4v88-pdb-bundle3.pdb	ABCDEFGHIJKLMNOPQRSTUVWXYZabcefghijklmnopqrstuvwxyz01	d
	4v88-pdb-bundle4.pdb	ABCDEFGHIJNOPQRS	KLM
	4v88-pdb-bundle5.pdb	ABCDEFGHIJKLMNOPQRSTUVWXYZabcdefghijklm	
4V8B	4v8b-pdb-bundle1.pdb	BCDEFGHIJKLMNOPQRSTU	AVWXY
	4v8b-pdb-bundle2.pdb	CDEFGHIJKLMNOPQRSTUVWXYZabcd	AB
	4v8b-pdb-bundle3.pdb	BCDEFGHIJKLMNOPQRSTU	AVWXY
	4v8b-pdb-bundle4.pdb	CDEFGHIJKLMNOPQRSTUVWXYZabcd	AB
4V8D	4v8d-pdb-bundle1.pdb	BCDEFGHIJKLMNOPQRSTU	AVWXY
	4v8d-pdb-bundle2.pdb	CDEFGHIJKLMNOPQRSTUVWXYZabcd	AB
	4v8d-pdb-bundle3.pdb	BCDEFGHIJKLMNOPQRSTU	AVWXY
	4v8d-pdb-bundle4.pdb	CDEFGHIJKLMNOPQRSTUVWXYZabcd	AB
4V8G	4v8g-pdb-bundle1.pdb	BCDEFGHIJKLMNOPQRSTUV	A
	4v8g-pdb-bundle2.pdb	CDEFGHIJKLMNOPQRSTUVWXYZabcde	AB
	4v8g-pdb-bundle3.pdb	BCDEFGHIJKLMNOPQRSTUV	A
	4v8g-pdb-bundle4.pdb	CDEFGHIJKLMNOPQRSTUVWXYZabcde	AB
4V8I	4v8i-pdb-bundle1.pdb	BCDEFGHIJKLMNOPQRSTUV	A
	4v8i-pdb-bundle2.pdb	CDEFGHIJKLMNOPQRSTUVWXYZabcde	AB
	4v8i-pdb-bundle3.pdb	BCDEFGHIJKLMNOPQRSTUV	A
	4v8i-pdb-bundle4.pdb	CDEFGHIJKLMNOPQRSTUVWXYZabcde	AB

**4V90**	4v90-pdb-bundle1.pdb	BCDEFGHIJKLMNOPQRSTUXYZabcdefgh	AVW
	4v90-pdb-bundle2.pdb	CDEFGHIJKLMNOPQRSTUVWX	AB
**4V9D**	4v9d-pdb-bundle1.pdb	BCDEFGHIJKLMNOPQRSTUXZabcdefgh	AVWY
	4v9d-pdb-bundle2.pdb	ABCDEFGHIJKPQRSTUVWXYZabcdefghijklmn	LMNO
	4v9d-pdb-bundle3.pdb	ABCDGHIJKLMNOPQRSTUVWXYZabcdefghi	EF
**4V9F**	4v9f-pdb-bundle1.pdb	ABCDEFGHIJKLMNOPQRSTUVWX	9
	4v9f-pdb-bundle2.pdb	YZ123456	
**4V9H**	4v9h-pdb-bundle1.pdb	DEFGHIJKLMNOPQRSTUVWX	ABC
	4v9h-pdb-bundle2.pdb	CDEFGHIJKLMNOPQRSTUVWXYZabcdefgh	AB
**4V9O**	4v9o-pdb-bundle1.pdb	BDEFGHIJKLMNOPQRSTUVWXYZabcdefgh	AC
	4v9o-pdb-bundle2.pdb	BCDEFGHIJKLMNOPQRSTUV	AW
	4v9o-pdb-bundle3.pdb	BCDEFGHIJKLMNOPQRSTUVWXYZabcde	A
	4v9o-pdb-bundle4.pdb	BCDEFGHIJKLMNOPQRSTUVW	A
	4v9o-pdb-bundle5.pdb	CDEFGHIJKLMNOPQRSTUVWXYZabcdef	AB
	4v9o-pdb-bundle6.pdb	BCDEFGHIJKLMNOPQRSTUVW	AX
	4v9o-pdb-bundle7.pdb	BCDEFGHIJKLMNOPQRSTUVWXYZabcd	A
	4v9o-pdb-bundle8.pdb	BCDEFGHIJKLMNOPQRSTUVW	A
**4V9P**	4v9p-pdb-bundle1.pdb	CDEFGHIJKLMNOPQRSTUVWXYZabcdef	AB
	4v9p-pdb-bundle2.pdb	BCDEFGHIJKLMNOPQRSTUVW	A
	4v9p-pdb-bundle3.pdb	CDEFGHIJKLMNOPQRSTUVWXYZabcde	AB
	4v9p-pdb-bundle4.pdb	BCDEFGHIJKLMNOPQRSTUVW	A
	4v9p-pdb-bundle5.pdb	CDEFGHIJKLMNOPQRSTUVWXYZabcdef	AB
	4v9p-pdb-bundle6.pdb	BCDEFGHIJKLMNOPQRSTUVW	A
	4v9p-pdb-bundle7.pdb	CDEFGHIJKLMNOPQRSTUVWXYZabcde	AB
	4v9p-pdb-bundle8.pdb	BCDEFGHIJKLMNOPQRSTUV	A
**4V9R**	4v9r-pdb-bundle1.pdb	BCDEFGHIJKLMNOPQRSTUX	AVW

	4v9r-pdb-bundle2.pdb	CDEFGHIJKLMNOPQRSTUVWXYZabcde	AB
	4v9r-pdb-bundle3.pdb	BCDEFGHIJKLMNOPQRSTUX	AVW
	4v9r-pdb-bundle4.pdb	CDEFGHIJKLMNOPQRSTUVWXYZabcde	AB
**4W2E**	4w2e-pdb-bundle1.pdb	DEFGHJKNOPQRSTUVWXYZ0123456789	ABx
	4w2e-pdb-bundle2.pdb	bcdefghijklmnopqrstuy	awv
**4W2F**	4w2f-pdb-bundle1.pdb	BCDEFGHIJKLMNOPQRSTU	AVWXY
	4w2f-pdb-bundle2.pdb	CDEFGHIJKLMNOPQRSTUVWXYZabcde	AB
	4w2f-pdb-bundle3.pdb	BCDEFGHIJKLMNOPQRSTU	AVWXY
	4w2f-pdb-bundle4.pdb	CDEFGHIJKLMNOPQRSTUVWXYZabcde	AB
**4W2G**	4w2g-pdb-bundle1.pdb	BCDEFGHIJKLMNOPQRSTU	AVWXY
	4w2g-pdb-bundle2.pdb	CDEFGHIJKLMNOPQRSTUVWXYZabcde	AB
	4w2g-pdb-bundle3.pdb	BCDEFGHIJKLMNOPQRSTU	AVWXY
	4w2g-pdb-bundle4.pdb	CDEFGHIJKLMNOPQRSTUVWXYZabcde	AB
**4W2H**	4w2h-pdb-bundle1.pdb	BCDEFGHIJKLMNOPQRSTU	AVWX
	4w2h-pdb-bundle2.pdb	CDEFGHIJKLMNOPQRSTUVWXYZabcde	AB
	4w2h-pdb-bundle3.pdb	BCDEFGHIJKLMNOPQRSTU	AVWX
	4w2h-pdb-bundle4.pdb	CDEFGHIJKLMNOPQRSTUVWXYZabcde	AB
**4W2I**	4w2i-pdb-bundle1.pdb	BCDEFGHIJKLMNOPQRSTU	AVWXY
	4w2i-pdb-bundle2.pdb	CDEFGHIJKLMNOPQRSTUVWXYZabcde	AB
	4w2i-pdb-bundle3.pdb	BCDEFGHIJKLMNOPQRSTU	AVWXY
	4w2i-pdb-bundle4.pdb	CDEFGHIJKLMNOPQRSTUVWXYZabcde	AB
**4WPO**	4wpo-pdb-bundle1.pdb	CDEFGHIJKLMNOPQRSTUVWXYZabcdefg	AB
	4wpo-pdb-bundle2.pdb	BCDEFGHIJKLMNOPQRSTUZ	AVWXY
	4wpo-pdb-bundle3.pdb	CDEFGHIJKLMNOPQRSTUVWXYZabcdefg	AB
	4wpo-pdb-bundle4.pdb	BCDEFGHIJKLMNOPQRSTUZ	AVWXY
**4WQF**	4wqf-pdb-bundle1.pdb	CDEFGHIJKLMNOPQRSTUVWXYZabcdefg	AB

4WQU	4wqf-pdb-bundle2.pdb	BCDEFGHIJKLMNOPQRSTUY	AVWX
	4wqf-pdb-bundle3.pdb	CDEFGHIJKLMNOPQRSTUVWXYZabcdefg	AB
	4wqf-pdb-bundle4.pdb	BCDEFGHIJKLMNOPQRSTUY	AVWX
	4wqu-pdb-bundle1.pdb	CDEFGHIJKLMNOPQRSTUVWXYZabcdefg	AB
	4wqu-pdb-bundle2.pdb	BCDEFGHIJKLMNOPQRSTUYZ	AVWX
	4wqu-pdb-bundle3.pdb	CDEFGHIJKLMNOPQRSTUVWXYZabcdefg	AB
	4wqu-pdb-bundle4.pdb	BCDEFGHIJKLMNOPQRSTUYZ	AVWX
4WQY	4wqy-pdb-bundle1.pdb	CDEFGHIJKLMNOPQRSTUVWXYZabcdefg	AB
	4wqy-pdb-bundle2.pdb	BCDEFGHIJKLMNOPQRSTUX	AVW
	4wqy-pdb-bundle3.pdb	CDEFGHIJKLMNOPQRSTUVWXYZabcdefg	AB
	4wqy-pdb-bundle4.pdb	BCDEFGHIJKLMNOPQRSTUX	AVW
4Y4O	4y4o-pdb-bundle1.pdb	CDEFGHIJKLMNOPQRSTUVWXYZabcde	AB
	4y4o-pdb-bundle2.pdb	BCDEFGHIJKLMNOPQRSTUV	A
	4y4o-pdb-bundle3.pdb	CDEFGHIJKLMNOPQRSTUVWXYZabcde	AB
	4y4o-pdb-bundle4.pdb	BCDEFGHIJKLMNOPQRSTUV	A
4Y4P	4y4p-pdb-bundle1.pdb	CDEFGHIJKLMNOPQRSTUVWXYZabcde	AB
	4y4p-pdb-bundle2.pdb	BCDEFGHIJKLMNOPQRSTU	AVWXY
	4y4p-pdb-bundle3.pdb	CDEFGHIJKLMNOPQRSTUVWXYZabcde	AB
	4y4p-pdb-bundle4.pdb	BCDEFGHIJKLMNOPQRSTU	AVWXY
4YBB	4ybb-pdb-bundle1.pdb	BCDEFGHIJKLMNOPQRSTUWXYZabcdefgh	AV
	4ybb-pdb-bundle2.pdb	ABCDEFGHKLMNOPQRSTUVWXYZabcdefghijklm	IJ
	4ybb-pdb-bundle3.pdb	CDEFGHIJKLMNOPQRSTUVWXYZabcdef	AB
4Z3Q	4z3q-pdb-bundle1.pdb	CDEFGHIJKLMNOPQRSTUVWXYZabcde	AB
	4z3q-pdb-bundle2.pdb	BCDEFGHIJKLMNOPQRSTU	AVWXY
	4z3q-pdb-bundle3.pdb	CDEFGHIJKLMNOPQRSTUVWXYZabcde	AB
	4z3q-pdb-bundle4.pdb	BCDEFGHIJKLMNOPQRSTU	AVWXY

4Z3S	4z3s-pdb-bundle1.pdb	CDEFGHIJKLMNOPQRSTUVWXYZabcde	AB
	4z3s-pdb-bundle2.pdb	BCDEFGHIJKLMNOPQRSTU	AVWXY
	4z3s-pdb-bundle3.pdb	CDEFGHIJKLMNOPQRSTUVWXYZabcde	AB
	4z3s-pdb-bundle4.pdb	BCDEFGHIJKLMNOPQRSTU	AVWXY
4Z8C	4z8c-pdb-bundle1.pdb	CDEFGHIJKLMNOPQRSTUVWXYZabcde	AB
	4z8c-pdb-bundle2.pdb	BCDEFGHIJKLMNOPQRSTUX	AVW
	4z8c-pdb-bundle3.pdb	CDEFGHIJKLMNOPQRSTUVWXYZabcde	AB
	4z8c-pdb-bundle4.pdb	BCDEFGHIJKLMNOPQRSTUX	AVW
